# Conserved Metabolic and Evolutionary Themes in Microbial Degradation of Carbamate Pesticides

**DOI:** 10.3389/fmicb.2021.648868

**Published:** 2021-07-07

**Authors:** Harshit Malhotra, Sukhjeet Kaur, Prashant S. Phale

**Affiliations:** Department of Biosciences and Bioengineering, Indian Institute of Technology-Bombay, Mumbai, India

**Keywords:** carbamate pesticide, toxicity, degradation, enzyme promiscuity, horizontal gene transfer, cellular compartmentalisation

## Abstract

Carbamate pesticides are widely used as insecticides, nematicides, acaricides, herbicides and fungicides in the agriculture, food and public health sector. However, only a minor fraction of the applied quantity reaches the target organisms. The majority of it persists in the environment, impacting the non-target biota, leading to ecological disturbance. The toxicity of these compounds to biota is mediated through cholinergic and non-cholinergic routes, thereby making their clean-up cardinal. Microbes, specifically bacteria, have adapted to the presence of these compounds by evolving degradation pathways and thus play a major role in their removal from the biosphere. Over the past few decades, various genetic, metabolic and biochemical analyses exploring carbamate degradation in bacteria have revealed certain conserved themes in metabolic pathways like the enzymatic hydrolysis of the carbamate ester or amide linkage, funnelling of aryl carbamates into respective dihydroxy aromatic intermediates, C1 metabolism and nitrogen assimilation. Further, genomic and functional analyses have provided insights on mechanisms like horizontal gene transfer and enzyme promiscuity, which drive the evolution of degradation phenotype. Compartmentalisation of metabolic pathway enzymes serves as an additional strategy that further aids in optimising the degradation efficiency. This review highlights and discusses the conclusions drawn from various analyses over the past few decades; and provides a comprehensive view of the environmental fate, toxicity, metabolic routes, related genes and enzymes as well as evolutionary mechanisms associated with the degradation of widely employed carbamate pesticides. Additionally, various strategies like application of consortia for efficient degradation, metabolic engineering and adaptive laboratory evolution, which aid in improvising remediation efficiency and overcoming the challenges associated with *in situ* bioremediation are discussed.

## Introduction

The sustenance of the ever-growing global population necessitates adequate crop production, therefore making the use of pesticides crucial to prevent loss in the agriculture sector. Furthermore, pesticides play an essential role in the prevention and control of potentially fatal vector-borne diseases like dengue, trypanosomiasis, leishmaniasis and chikungunya, especially in high-risk tropical regions. Up until the 1940s, inorganic substances like sulphuric acid and sodium chlorate or naturally occurring organic compounds like petroleum oil, naphthalene and creosote were used as pesticides (Unsworth, 2010). However, these compounds were inefficient and World War II imposed an urgency to significantly increase crop production, leading to the development of synthetic pesticides like dichloro-diphenyl-trichloroethane (DDT), Aldrin, Dieldrin, 2,4-dichlorophenoxy acetic acid (2,4-D) and parathion. Consequently in the 1960s, the hazardous effects of widespread use of pesticides like insect resistance, toxicity to non-target organisms and ecological disturbance became evident (Carson, 1962), leading to the development of more selective and safe alternatives. However, pesticide use continued for ensuring food productivity and public health, paving the way for collateral hazards (Bernardes et al., 2015).

On the basis of chemical properties, pesticides are classified into organochlorines, organophosphorus, carbamates, pyrethroids, amides, anilins, and azotic heterocyclic compounds (Özkara et al., 2016). Amongst these, the carbamate class of pesticides are derivatives of carbamic acid and characterised by a carbamate ester bond as functional group (which is linked to either aromatic or oxime moiety). These compounds act as reversible inhibitors of the enzyme, acetylcholinesterase (AChE) of the nervous system. They show broad-spectrum activity against insects, nematodes, molluscs as well as arachnids. Furthermore, they function as herbicides by inhibiting the photosynthesis electron transport chain and fungicides by binding spindle microtubules and causing nuclear division blockade (Singh et al., 2016; Pujar et al., 2019; Mishra et al., 2021b). Examples of some of the commonly used carbamate pesticides include fenobucarb, carbofuran, phenmedipham, carbendazim, aldicarb, methomyl, oxamyl, propoxur and Carbaryl (Table 1).

**TABLE 1:** Physico-chemical properties of various carbamate pesticides and microbes involved in their degradation.

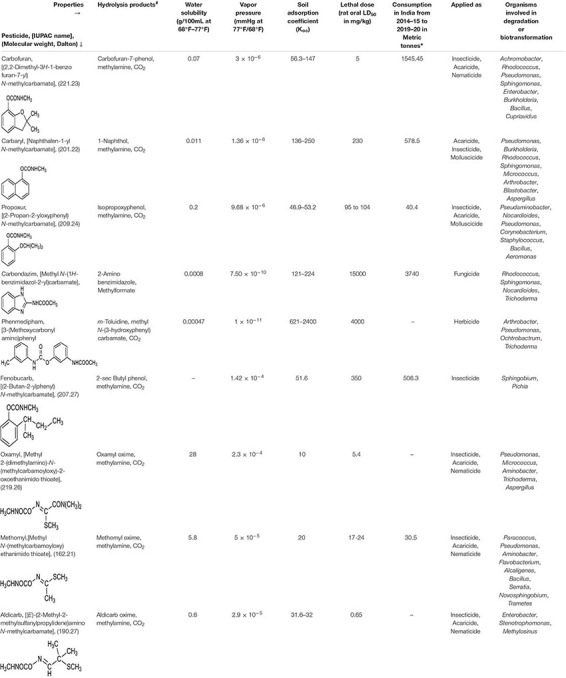

# Enzymatic or alkaline hydrolysis. *Data obtained from: http://ppqs.gov.in/statistical-database, latest accessed on 30th December 2020.

The widespread and repeated application of carbamate pesticides has led to their distribution into various compartments of the biosphere, thus imposing a selection pressure on the microbiota to evolve detoxification and/or degradation pathways. Various bacterial and fungal species have been reported to degrade carbamates and utilise them as sole carbon and nitrogen source. Here we review the toxicity imposed by carbamate pesticides as well as recent leads in the evolution and adaptation of various metabolic pathways at the biochemical and genetic level.

## Environmental Fate

**FIGURE 1:**
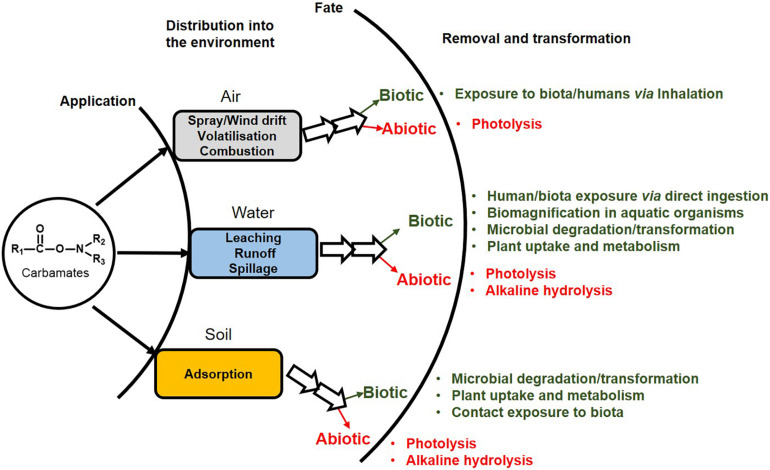
Mode of entry of carbamate pesticides into the environment and their fate.

Of the total carbamates applied as pesticides in the agriculture sector, only a minor fraction impacts the target organisms, whereas the rest of it is distributed into the environment, harming non-target biota and leading to ecological imbalance. Processes that mainly contribute to distribution and persistence of these compounds include leaching, adsorption, run-off, volatilisation, and partial degradation (by both biotic and abiotic factors) (Arias-Estévez et al., 2008; Lin et al., 2020; Figure 1). Apart from biotic and abiotic factors, the presence of co-contaminants like heavy metals might impact the persistence of these compounds in the environment by altering their adsorption, bioavailability, redox state as well as the toxicity. Further, heavy metals might interact with the biota at the site of contamination, to either enhance or inhibit degradation (Zhang et al., 2020). Since carbamates differ widely in chemical properties like water solubility, vapor pressure, photostability and soil adsorption coefficient (K_oc_), the environmental fate of each individual pesticide is variable (Table 1).

Highly water-soluble carbamates like methomyl, oxamyl and aldicarb are more prone to run-off and leaching into groundwater and water bodies (Malato et al., 2000; Choquette, 2014; Lin et al., 2020). Whereas, aromatic-based carbamate pesticides can adsorb onto soil particles and enter water bodies. The organic content of the soil is a major determinant of carbamate adsorption and certain compounds like Carbaryl and carbofuran show moderate to high Sorption in soil (Leenheer and Ahlrichs, 1971; Cáceres et al., 2019). Apart from presence in water and soil, carbamates are also detected in the air due to spray drift or volatilisation (Ferreira and Seiber, 1981; Table 1).

In order to determine the fate of these pesticides, various techniques are employed in quantitative and qualitative estimation from environmental samples. Gas chromatography (GC) and high-performance liquid chromatography (HPLC), coupled with mass spectrometry (MS) or other methods of detection (like UV-Vis/fluorescence) have primarily been applied in these analyses. However, as most carbamates are polar and thermally unstable, derivatisation is often required for GC analysis (Wu et al., 2009). For example, GC–MS (0.25 μm film thickness; 10°C/min raising rate) upon liquid-phase microextraction (LPME) and on-column derivatisation has been used for the determination of promecarb, propham, Carbaryl, methiocarb and chlorpropham residues in tap and waste water (Zhang and Lee, 2006). Similarly, a highly sensitive method for determination of carbofuran, tsumacide, isoprocarb and pirimicarb in water samples has been developed by combination of GC/MS (0.25 μm film thickness; 5°C/min raising rate) and dispersive liquid–liquid microextraction (Chen H. et al., 2010). Determination of Carbaryl, carbofuran, metolcarb, isoprocarb and ethiofencarb could be carried out in surface waters by GC/MS (0.25 μm film thickness; 5–30°C/min raising rate) upon derivatisation with 9-xanthydrol (Yang and Shin, 2013). Wu et al. (2009) employed HPLC, coupled with diode array detection in order to determine carbofuran, diethofencarb, Carbaryl and pirimicarb in water samples, upon dispersive liquid–liquid microextraction. Recently, isotope dilution liquid chromatography, coupled with MS, has been applied for the determination of Carbaryl, carbofuran and carbendazim in vegetables (Ahn et al., 2021).

A variety of both biotic and abiotic processes are responsible for degradation or transformation of carbamate pesticides in the environment. Abiotic routes include direct or indirect photodegradation, alkaline hydrolysis, volatilisation and adsorption, amongst others (Faust and Gomaa, 1972; de Bertrand and Barceló, 1991; Raut-Jadhav et al., 2016; Tomašević et al., 2019). Biotic routes include microbial degradation as the primary route for their complete removal by bacterial and fungal species. Various microbial species belonging to the genera *Pseudomonas*, *Stenotrophomonas*, *Micrococcus*, *Enterobacter*, *Nocardioides*, *Pseudaminobacter*, *Serratia*, *Mucor*, *Trametes*, *Trichoderma*, *Pichia* and *Aspergillus*, amongst others, have been reported to be involved in the transformation or degradation of carbamate pesticides (Mustapha et al., 2019).

## Toxicity of Carbamates

Toxicity is mediated through both cholinergic and non-cholinergic mode. Due to the presence of AChE and the related enzyme butyrylcholinesterase in mammals (including humans), birds, fish, reptiles and insects, carbamate pesticides also impact non-target organisms, leading to ecological imbalance (Fukuto, 1990). They are reported to carry out reversible carbamylation of active site amino acid residue serine of AChE, leading to accumulation of acetylcholine at synapses and neuromuscular junctions, causing “cholinergic crisis.” AChE, under normal conditions, catalyses the hydrolysis of acetylcholine (neurotransmitter) to acetic acid and choline, leading to cessation of signalling (Waseem et al., 2010; Silberman and Taylor, 2020).

Apart from hazards emerging from cholinergic excess, carbamate pesticides and their metabolites act as endocrine disruptors by potentiating or antagonizing the activity of steroid hormones, thus disrupting steroidogenesis and thyroid function. Carbamate pesticides (and their metabolites) mimic various hormones and bind to their receptors, therefore affecting the expression of the responsive genes. The estrogen receptor α and β (ERα and ERβ) are estrogen binding transcription factors that regulate steroid hormone mediated gene expression (Kalaitzidis and Gilmore, 2005). Similar receptors exist for androgens and progesterone. Various carbamates like oxamyl, methomyl and Carbaryl, have been shown to act as weak agonists of the human estrogen receptor (hER) and human progesterone receptor (hPR) in breast (MCF-7) and endometrial (Ishikawa) cancer cells (Klotz et al., 1997). Carbaryl and methiocarb (and their hydrolysis products) were shown to exhibit *in vitro* ERα and ERβ agonistic activity. Furthermore, these compounds exhibited antiandrogenic activity (Tange et al., 2016). Pirimicarb, another carbamate pesticide, has been reported to decrease ERα mRNA levels (Grünfeld and Bonefeld-Jorgensen, 2004). Moreover, the expression of the androgen receptor gene was increased in rats upon exposure to carbendazim, a carbamate family fungicide (Hsu et al., 2011). These compounds have also been shown to disrupt the levels of various steroid hormones. For example, carbendazim exposure has been reported to be associated with increased estrogen levels (Morinaga et al., 2004), Carbaryl has been reported to inhibit progesterone biosynthesis in primary human granulose–lutein cells (Cheng et al., 2006) and carbofuran has been shown to disrupt the progesterone, estradiol, cortisol, and testosterone levels in rodents (Goad et al., 2004).

The metabolism of carbamate pesticides imposes an oxidative stress due to generation of either reactive toxic metabolites or reactive oxygen species (ROS). These toxic intermediates interact with macromolecules like proteins, lipids as well as DNA, leading to cytotoxicity (Dhouib et al., 2016). For example, *N*-nitrosocarbofuran, a gastric metabolite of carbofuran, has been postulated to produce an O^6^MeG DNA adduct, thus acting as a mutagen and carcinogen (Lee et al., 2004). Similarly, *N*-nitroso propoxur induced chromosome aberrations and sister-chromatid exchanges have been reported to occur in Chinese hamster ovary (CHO-W8) cells as a result of O^6^MeG DNA lesions (Lin et al., 2007). Apart from DNA, carbofuran-albumin and immunoglobulin adducts have been found to occur in human sera (Rehman et al., 2016). Lipid peroxidation is another significant example of ROS mediated macromolecular damage; caused by the interaction of oxygen free radicals (generated upon pesticide exposure) with polyunsaturated fatty acids constituting the cell membrane (Banerjee et al., 2001). Exposure to various carbamate pesticides like carbofuran (Rai and Sharma, 2007), aldicarb (Yarsan et al., 1999), oxamyl (Prezenská et al., 2019) and methomyl (Mansour et al., 2009) has been reported to stimulate and enhance lipid peroxidation by generation of ROS.

Further, exposure to carbamate pesticides has been reported to enhance the activity of various cellular antioxidant enzyme systems; a defence mechanism against these pro-oxidant compounds. However, prolonged exposure depletes these essential antioxidant systems, leading to cellular damage (Ndonwi et al., 2019). For example, exposure to carbofuran has been reported to cause significant elevation in activity of the antioxidant enzymes, superoxide dismutase and catalase, in rat brain and liver (Rai and Sharma, 2007). Similarly, in CHO-K1 cells, exposure to propoxur and aldicarb (and its metabolites aldicarb sulfone and aldicarb sulfoxide) is associated with an increase in the activity of glutathione peroxidase, glutathione reductase and glutathione transferase, as well as depletion of the intracellular reduced glutathione (involved in free-radical scavenging) content (Maran et al., 2009). Chronic propoxur exposure in rats is also associated with increased activities of the antioxidant enzymes superoxide dismutase and catalase, glutathione peroxidase, glutathione reductase, and glutathione S-transferase in the blood (Seth et al., 2001).

ROS also affects the mitochondria, which plays an important role in early and later fetal development stages (Coffman et al., 2009; Le Belle et al., 2011). Further damage to mitochondrial DNA and other macromolecules by ROS leads to prenatal mitochondrial impairment, resulting in embryotoxicity. Besides ROS mediated damage, carbamates have been reported to interact with mitochondrial translocator and other proteins, which results in the disruption of mitochondrial lipid metabolism and function (Leung and Meyer, 2019). The ROS generated upon carbamate exposure causes toxicity to immune cells like B, T, and NK cells, thus compromising the hosts immunity (Dhouib et al., 2016). Cholinergic excess has been reported to increase IL-2 signalling in B- and T- cells as well as inflammatory responses in macrophages (Banks and Lein, 2012). The interaction of steroid hormones with the receptors in immune cells is essential for their functioning. Carbamates are also known to interact with the hypothalamic–pituitary–adrenal axis (HPA axis) and disrupt gonadotropin-releasing hormone (GnRH) biosynthesis and GnRH mediated signalling in immune cells (Mokarizadeh et al., 2015).

Various reports have evaluated the *in vitro* toxicity of carbamate compounds in mammalian cells. For example, the lysosomal function and mitochondrial integrity of CHO-K1 cells were evaluated upon exposure to varying concentrations of seven carbamate pesticides, in presence or absence of foetal calf serum. Aldicarb was found to be the most toxic (MTT_50_ = 164 ± 29 μM), followed by propoxur (MTT_50_ = 161 ± 39 μM), aldicarb sulfone (MTT_50_ = 211 ± 41 μM) and aldicarb sulfoxide (MTT_50_ = 1162 ± 94 μM). Whereas exposure to pirimicarb, thiobencarb and benfuracarb did not show a concentration-dependent cytotoxic effect (José Ruiz et al., 2006). Similarly, exposure to 10–100 μg/ml of carbofuran showed a concentration-dependent increase in sister chromatid exchange frequency and micronuclei induction in CHO-K1 cells, indicating its genotoxic potential. Further, exposure to 100 μg/ml of carbofuran was found to significantly decrease cell growth and viability of the CHO-K1 cell line (Soloneski et al., 2008). Exposure to carbofuran has also been shown to induce genotoxicity and cytotoxicity in cat (*Felis catus*) fibroblast cells. The LC_50_ value was estimated to be 0.42 mM and maximum DNA damage was induced at 1.08 mM (Chandrakar et al., 2020). Pirimicarb (10–300 μg/ml) has been reported to induce chromosomal aberrations, chromatid and isochromatid-type breaks in CHO-K1 cells in a concentration-dependent manner, while sister-chromatid exchanges were observed at concentrations of 100–200 μg/ml and a delay in cell-cycle kinetics was observed in the 100–300 μg/ml range (Soloneski and Larramendy, 2010). Pirimicarb (10–300 μg/ml) has also been shown to display a concentration-dependent increase in micronucleus induction and decrease in cell viability (CHO-K1 cells; Soloneski et al., 2015). Exposure to 4.09–1046.4 μM of carbendazim displayed a concentration-dependent decrease in cell viability of A549 alveolar (epithelial) cell monolayer (IC_25_ = 12.5 μM). Cell cycle arrest in G2/M phase was observed, corresponding to DNA fragmentation. In addition to monolayer, a 3D alveolar epithelial model was constructed and exposed to carbendazim in the air-liquid interface. This model displayed a significant increase in ROS formation, decreased mitochondrial activity, increased caspase activity and alterations in α-tubulin levels, leading to decreased viability (Tollstadius et al., 2019). Recent studies have evaluated the impact of carbamate exposure on human endothelial cells (Saquib et al., 2021a,b). Exposure to methomyl, Carbaryl, metalaxyl, and pendimethalin (500 and 1,000 μM) significantly affected the proliferation of human umbilical vein epithelial cells and induced ROS generation, DNA damage and apoptosis (Saquib et al., 2021b). Similarly, exposure to carbofuran (100–1,000 μM) induced oxidative stress, mitochondrial membrane hyperpolarisation, DNA damage, apoptosis and significantly reduced the human umbilical vein epithelial cell viability at concentrations of 500 and 1,000 μM (Saquib et al., 2021a). Toxicity of carbamate pesticides to immune cells has also been evaluated *in vitro* at varying concentrations. A concentration dependent increase was observed in the frequency of sister chromatid exchanges in human lymphocyte cultures upon exposure to 250–750 mg/L of a 90% methomyl based formulation, while 1,000 mg/L caused cellular death (Valencia-Quintana et al., 2016). Similarly, carbofuran-treated human lymphocyte cultures displayed chromosomal aberrations and an LD_50_ of 18 μM was reported (Naravaneni and Jamil, 2005). Carbaryl (0–40 μM) has been reported to induce apoptosis in Jurkat human T-lymphocytes in a time- and dose-dependent manner (Li Q. et al., 2015).

**FIGURE 2:**
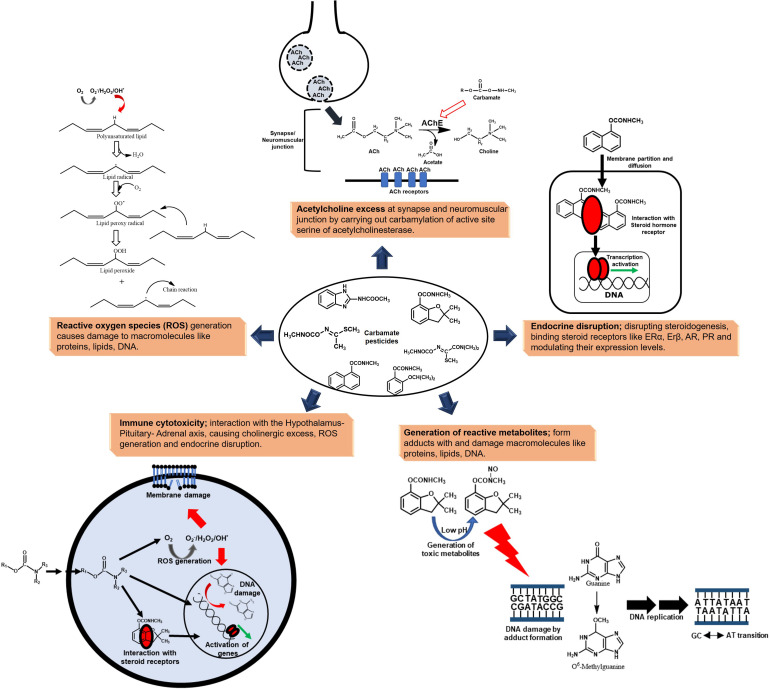
Toxicity of carbamate pesticides manifested at various levels to humans and non-target biota.

Hence, various modes of action contribute to the mutagenic, teratogenic, carcinogenic and genotoxic effects upon carbamate exposure (Figure 2). Therefore, the removal of these compounds from the environment is very essential.

## Bacterial Metabolism of Carbamate Pesticides

Microbial degradation of carbamates involves either partial transformation or complete mineralisation/utilisation (Table 1). Prokaryotes share certain common features in degradation of carbamate pesticides. For example, the first step involves the hydrolysis of the carbamate ester or amide linkage, leading to the formation of hydrolysis product, methylamine and carbon dioxide (CO_2_). Methylamine is utilised both as a carbon and nitrogen source by C1 metabolism. The diversity in degradation occurs primarily due to processing of the remaining carbon skeleton. Carbamates like propoxur, Carbaryl, carbofuran and carbendazim, which harbour aromatic nuclei are degraded through the formation of the respective dihydroxy intermediates, like catechols (Trivedi et al., 2016; Long et al., 2020; Mishra et al., 2020). Whereas, oxime pesticides like aldicarb, oxamyl and methomyl form respective oximes upon hydrolysis which are utilised through unknown routes (Kazumi and Capone, 1995; Rousidou et al., 2016; Lin et al., 2020). Apart from traditionally used carbamates, pesticides belonging to other classes might harbour a carbamate moiety. For example, the degradation of pyraclostrobin (a strobilurin fungicide harbouring carbamate moiety) has been reported to proceed *via* hydrolysis and decarboxylation of the tertiary amine group to a primary amine, possibly by the action of a carboxylesterase (Chen et al., 2018).

Various techniques can be employed for the elucidation of carbamate degradation pathways in bacteria. The intermediates involved in carbamate degradation are identified by thin layer chromatography, GC, HPLC and MS, or a combination of these. The ability of the strain to grow on these intermediates as sole carbon sources can also be examined. Further, monitoring the enzyme activity in cell-free extract and whole cells can aid in identification of the pathway enzymes. Whole-cell respiration studies examine the oxygen uptake on various substrates and intermediates, and serve as an essential tool for elucidation of steps involved in carbamate degradation. Additionally, genome analyses and annotation identify putative genes (encoding enzymes) involved in the degradation of these compounds (Swetha and Phale, 2005; Nguyen et al., 2014; Yan et al., 2018; Pujar et al., 2019; Long et al., 2020).

The metabolic pathways for carbamate degradation are orchestrated by enzymes primarily belonging to the hydrolase and oxidoreductase class. Hydrolases participate in the first step of carbamate degradation which involves hydrolysis of ester or amide bond of the carbamate moiety. For example, the Carbaryl hydrolases (CH), CehA and McbA from *Rhizobium* sp. AC100 and *Pseudomonas* sp. C5pp, respectively, belong to the esterase family (Hashimoto et al., 2002; Trivedi et al., 2016). Apart from carbamates, esterases have been found to participate in the degradation and detoxification of ester-moiety containing organophosphate as well as pyrethroid pesticides, and follow a common hydrolysis mechanism involving a catalytic triad (Bhatt et al., 2021b). While, the CH, CahA from *Arthrobacter* sp. RC100 belongs to the amidase family (Hashimoto et al., 2006). Carbamates like Carbaryl, phenmedipham and propoxur harbour an aromatic moiety which makes them highly hydrophobic and resistant to degradation. Bacteria overcome this barrier by the oxygenation (hydroxylation) of the aromatic nucleus, thus increasing its oxidation status (Phale et al., 2020). These reactions are catalysed by oxygenases, which can be further classified into monoxygenases/hydroxylases and dioxygenases (ring-hydroxylating and ring-cleaving). The aromatic hydrolysis product is further hydroxylated by a monooxygenase to make it more susceptible for degradation. Apart from activation by ring hydroxylation, monooxygenases are also involved in the conversion of monohydroxylated aromatic intermediates into respective dihydroxy intermediates. For example, salicylate-1-hydroxylase in Carbaryl degradation pathway converts salicylate to catechol, whereas salicylate-5-hydroxylase converts it to gentisate (Phale et al., 2019). This conversion step to dihydroxy- intermediates can also be catalysed by dioxygenases as in the case of benzoate-1,2-dioxygenase in the carbendazim degradation pathway and toluidine dioxygenase in the phenmedipham degradation pathway (Pujar et al., 2019; Long et al., 2020). Dioxygenases also carry out the aromatic ring-cleavage of the respective catechols. These ring-cleaved intermediates are further converted into central carbon pathway metabolites and assimilated as the sole source of carbon and energy.

**FIGURE 3:**
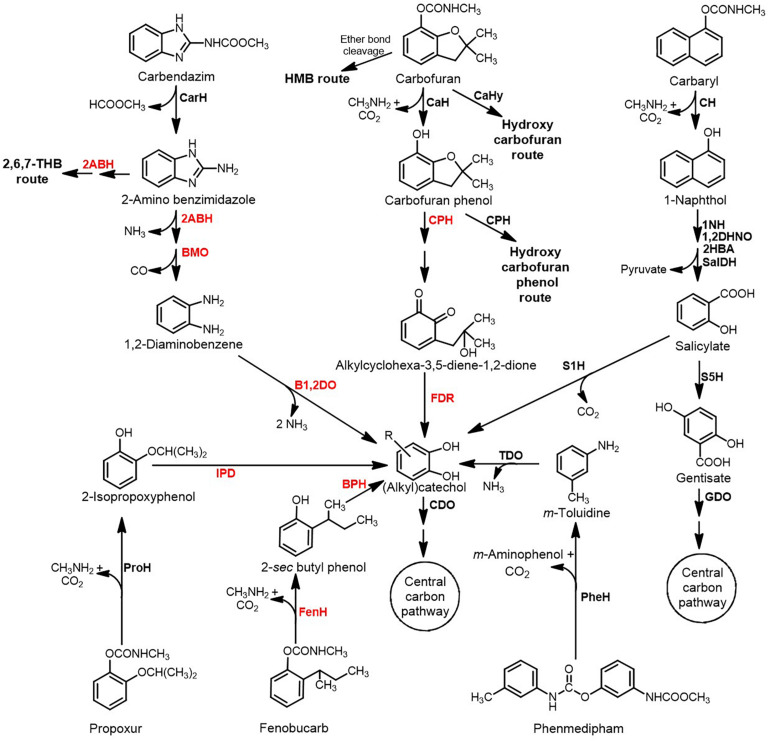
Metabolic pathways for the degradation of aromatic ring-based carbamate pesticides. Various key enzymes involved are depicted in bold: CarH, Carbendazim hydrolase; 2ABH, 2-Aminobenzimidazole hydroxylase; BMO, Benzimidazolone monooxygenase; B1,2DO, Benzoate-1,2-dioxygenase; CaH, Carbofuran hydrolase; CaHy, Carbofuran hydroxylase; CPH, Carbofuran phenol hydroxylase; FDR, Flavin-dependent reductase; CH, Carbaryl hydrolase; 1NH, 1-Naphthol 2-hydroxylase; 1,2DHNO, 1,2-Dihydroxynaphthalene dioxygenase; 2HBA, 2-Hydroxybenzalpyruvate aldolase; SalDH, Salicylaldehyde dehydrogenase; S1H, Salicylate1-hydroxylase; S5H, Salicylate 5-hydroxylase; GDO, Gentisate dioxygenase; ProH, Propoxur hydrolase; IPD, Isopropoxyphenol *O*-dealkylase; FenH, Fenobucarb hydrolase; BPH, 2-*sec* Butylphenol hydroxylase; PheH, Phenmedipham hydrolase; TDO, Toluidine dioxygenase; and CDO, Catechol dioxygenase. Enzymes in the red font indicate the activity was reported at the biotransformation or annotation at the genome level; while those in black font indicates enzyme activities measured from the cell-free extract and some of them have been purified and characterised. HMB indicates 2-hydroxy-3-(3-methylpropan-2-ol) benzene-*N*-methylcarbamate.

Apart from oxygenases, dehydrogenases also participate in redox reactions. For example, the salicylaldehyde generated in Carbaryl degradation pathway is converted to salicylate by NAD^+^-dependent salicylaldehyde dehydrogenase (Swetha and Phale, 2005). Other classes of enzymes like lyases and isomerases also play an important role in processing of the aliphatic carbon skeleton upon de-aromatisation by ring cleavage (Nguyen et al., 2014; Trivedi et al., 2016; Figure 3). For example, 2-hydroxychromene 2-carboxyl isomerase catalyses the conversion of 2-hydroxychromene 2-carboxylate to 2-hydroxybenzalpyruvate, which is acted upon by 2-hydroxybenzalpyruvate aldolase to form salicylaldehyde in the Carbaryl degradation pathway (Phale et al., 2019).

### Carbamate Pesticides as the Carbon Source

#### Carbofuran

Carbofuran [(2,2-Dimethyl-3*H*-1-benzofuran-7-yl) *N*-methyl carbamate] is a highly toxic, broad spectrum pesticide that acts as a nematicide, acaricide and insectide. Carbofuran degradation pathways are diverse and have been deciphered in *Sphingomonas* sp. CDS-1, *Novosphingobium* sp. KN65.2, *Sphingobium* sp. CFD-1, *Cupriavidus* sp. ISTL and *Pseudomonas* sp. 50432 (Figure 3; Mishra et al., 2020). The first step in carbofuran hydrolysis involves the cleavage of ester bond and is catalysed by carbofuran hydrolase (CaH; Mcd or CehA). The metallohydrolase, Mcd is a Mn^2+^-dependent esterase that was first identified in *Achromobacter* sp. WM111 and has been extensively characterised (Tomasek and Karns, 1989; Naqvi et al., 2009). Mcd has also been detected in *Rhodococcus* and *Pseudomonas* spp. and is seldom associated with carbofuran mineralising organisms (Öztürk et al., 2016). On the other hand, the CH, CehA was first identified in *Rhizobium* sp. AC100, but has been reported to be inefficient in hydrolysing carbofuran. However, its homologs have been identified in carbofuran degrading bacteria like *Sphingomonas* sp. CDS-1 and *Novosphingobium* sp. KN65.2 (Yan et al., 2018).

In *Novosphingobium* sp. KN65.2, the initial step involves hydrolysis of the carbamate linkage to form carbofuran phenol, methylamine and CO_2_ (Figure 3; Nguyen et al., 2014). The phenol undergoes *ipso*-hydroxylation and furanyl ring-cleavage by the action of carbofuran phenol hydroxylase (CfdC) to produce 3-(2-hydroxy-2-methylpropyl) cyclohexa-3,5- diene-1,2-dione). This intermediate is further reduced to the catechol, 3-(2-hydroxy-2-methylpropyl) benzene-1,2-diol by the action of a (yet unknown) flavin dependent reductase. The dioxygenase CdfE then causes the dearomatisation of the catechol by ring-cleavage to form (*2E,4Z*)-2,8-dihydroxy-8-methyl-6-oxonona-2,4-dienoic acid. This aliphatic intermediate is hydrolysed to 3-hydroxy-3-methyl butanoic acid and 2-oxopent-4-enoate, which are then converted to central carbon metabolites (Figure 3). Apart from this route, a parallel carbofuran side-chain hydroxylation route has also been proposed in this organism (Nguyen et al., 2014). The carbofuran degradation pathway and enzymes in strain KN65.2 have been proposed on the basis of genome annotation, transcriptional analysis, plasposon mutagenesis and spent media analysis (Nguyen et al., 2014). Degradation of carbofuran in *Cupriavidus* sp. ISTL7 has also been proposed to follow the same hydrolytic degradation route through the formation of carbofuran phenol and further to catechol, 3-(2-hydroxy-2-methylpropyl) benzene-1,2-diol (Gupta et al., 2019). Formation of a red colour intermediate by condensation of several carbofuran degradation metabolites has been reported in *Sphingomonas* sp. SB5 and strain KN65.2 (Kim et al., 2004; Park et al., 2006).

Carbofuran degradation in *Sphingobium* sp. CFD-1 proceeds through the formation of carbofuran phenol. The phenol can further be hydroxylated to 4-hydroxycarbofuran phenol by carbofuran phenol hydroxylase, which can be interconverted to a dione (Jiang et al., 2020a; Figure 3). This 4-hydroxycarbofuran phenol further, through yet unknown metabolic steps, yields central carbon pathway intermediates. The esterase-type CaH, CehA from strain CFD-1 has been purified and characterised upon heterologous expression. Four histidine residues, His313, His315, His453 and His495 were found to play an important role in the hydrolysis of carbofuran (Jiang et al., 2020a). Whereas in *Sphingomonas* sp. CDS-1, carbofuran and carbofuran phenol are transformed, but the bacterium failed to grow on either of them. The transformation of carbofuran phenol has been shown to be carried out by the hydroxylase-reductase pair CfdCX (Yan et al., 2018). Both the proteins, CfdC and CfdX were individually purified and characterised. CfdX was found to be the reductase protein that required NADH (but not NADPH) as cofactor and FMN/FAD as a prosthetic group. CfdC was reduced flavin mononucleotide or reduced flavin adenine dinucleotide-dependent monooxygenase that required CfdX to function. Together the CfdCX pair was found to carry out the hydroxylation of carbofuran phenol at the *para* position of the benzene ring (Yan et al., 2018).

In *Novosphingobium* sp. FND-3, carbofuran degradation has been proposed by three distinct routes: i) hydrolysis of the carbamate ester linkage to form carbofuran phenol, ii) hydrolysis of the ether linkage in the furanyl ring to form 2-hydroxy-3-(3-methylpropan-2-ol) benzene-*N*-methylcarbamate and iii) oxidation of the aromatic nucleus to form hydroxycarbofuran (hydroxycarbofuran route; Figure 3; Yan et al., 2007). *Pseudomonas* sp. 50432 harbours both oxidative and hydrolytic pathways for carbofuran degradation resulting in the formation of 5-hydroxycarbofuran and carbofuran phenol, respectively (Chaudhry et al., 2002). *Rhodococcus* sp. TEI shows a similar oxidative mechanism involving the conversion of carbofuran to 5-hydroxycarbofuran (Figure 3; Mishra et al., 2020).

#### Carbaryl

Carbaryl (Naphthalen-1-yl *N*-methylcarbamate) is a naphthalene-based carbamate pesticide. Being sparingly soluble in water, it is reported to be absorbed strongly onto soil particles. Large number of bacterial isolates have been reported to either transform Carbaryl into 1-naphthol (a highly toxic intermediate), or mineralise it completely (Phale et al., 2019; Figure 3). The first step involves hydrolysis of Carbaryl to 1-naphthol, methylamine and CO_2_ by CH, which can either be an esterase or an amidase. Examples of esterase type of CH include CehA from *Rhizobium* sp. AC100 and McbA from *Pseudomonas* sp. C5pp and *Pseudomonas putida* XWY-1 (Hashimoto et al., 2002; Trivedi et al., 2016; Zhu et al., 2019) whereas amidase type CH has been reported in *Arthrobacter* sp. RC100 (Hashimoto et al., 2006). McbA from strain XWY-1 was purified and characterised. This protein harboured three histidine residues (His467, His477 and His504) in the predicted polymerase/histidinol phosphatase-like domain, that were crucial for hydrolysis (Zhu et al., 2018). The 1-naphthol generated undergoes hydroxylation by 1-naphthol 2-hydroxylase (1NH) to yield 1,2-dihydroxynaphthalene. 1NH has been purified and characterised from *Pseudomonas* sp. C4, C5pp and C6 (Swetha et al., 2007; Sah and Phale, 2011; Trivedi et al., 2014). In all three strains, this enzyme was found to be a single-component homodimeric protein belonging to NAD(P)H-dependent external flavin monooxygenase group. In strain C5pp, 1NH (McbC) has tightly bound FAD and shows equal efficiency for NADH and NADPH as a cofactor (Trivedi et al., 2014). Recently, a two-component 1NH (CehC1C2) from *Rhizobium* sp. X9 has been purified and characterised. CehC1 functions as an FMNH_2_ or FADH_2_-dependent monooxygenase, while CehC2 is a reductase that utilises NADH to reduce FAD or FMN. Compared to the single component 1NH (McbC), the two-component 1NH (CehC1C2) showed significantly lower hydroxylation activity with 1-naphthol, but displayed a broader substrate specificity (Zhou et al., 2020).

The 1,2-dihydroxynaphthalene generated undergoes *meta* ring-cleavage by 1,2- dihydroxynaphthalene dioxygenase, forming an unstable intermediate which converts to 2-hydroxychromene-2-carboxylic acid, which undergoes isomerisation to form *trans*-hydroxybenzylidene pyruvate (Figure 3). This intermediate is metabolised to salicylaldehyde and pyruvate by the action of a hydratase-aldolase. Salicylaldehyde is oxidised to salicylate by the action of NAD^+^-dependent salicylaldehyde dehydrogenase (Phale et al., 2019). This enzyme has been purified from Carbaryl-degrading *Pseudomonas* sp. C6 and was found to be a homotrimer and showed broad substrate specificity on mono- as well as di-aromatic aldehydes, in addition to salicylaldehyde (Singh et al., 2014). In certain bacteria like *Pseudomonas* sp. C5pp, *Rhodococcus* sp. NCIB 12038, *Pseudomonas* sp. NCIB 12043 and *P. putida* XWY-1, salicylate is metabolised to gentisate by salicylate-5-hydroxylase (S5H), whereas in others like *Pseudomonas* sp. NCIB 12042, salicylate-1-hydroxylase (S1H) acts to form catechol (Phale et al., 2019; Figure 3). S5H has been purified from the naphthalene degrader *Ralstonia* sp. U2 and was found to be a multi-subunit complex that utilises NADPH/NADH as a co-factor, FAD as a prosthetic group and consists of oxygenase, ferredoxin and ferredoxin reductase subunits/components (Fang and Zhou, 2014). S5H has also been reported to be a homotetramer in *Rhodococus erythropolis* S-1, and requires NADH as a cofactor and FAD as a prosthetic group (Suemori et al., 1993). On the other hand, S1H can either be monomeric (Takemori et al., 1974), dimeric (White-Stevens and Kamin, 1972) or multi-subunit, harbouring separate oxygenase, ferredoxin and reductase components (Jouanneau et al., 2007). Like S5H, S1H also requires NADPH/NADH as a cofactor and FAD as a prosthetic group.

Catechol or gentisate undergo ring-cleavage by the action of catechol dioxygenase or gentisate dioxygenase to yield aliphatic intermediates, which are funnelled into central carbon metabolic pathways (Phale et al., 2019). Certain isolates like *Burkholderia* sp. C3 have the ability to metabolise salicylate through both routes (Seo et al., 2013).

#### Carbendazim

Carbendazim [Methyl *N*-(1*H*-benzimidazol-2-yl) carbamate] is a broad-spectrum fungicide to control *Ascomycetes*, *Fungi imperfecti* and *Basidiomycetes* on crops like banana, citrus and strawberry. It is highly persistent in the soil due to presence of hydrophobic benzimidazolic ring. It is unique in its mode of action amongst various carbamate pesticides as it binds to spindle microtubules and causes nuclear division blockade (Singh et al., 2016). Degradation of carbendazim in bacteria proceeds by the hydrolysis of methyl carbamate side-chain to generate 2-aminobenzimidazole and methyl formate. The carbendazim hydrolase, MheI from *Microbacterium* sp. djl-6F has been purified and characterised. The enzyme does not require any co-factor and has two cysteine residues, Cys16 and Cys222, involved in hydrolysis (Lei et al., 2017). Similarly, the carbendazim hydrolase, MheI from *Mycobacterium* sp. SD-4 was characterised (Zhang Y. et al., 2017). A very similar, but extracellular carbendazim hydrolase, MheI from *Nocardioides* sp. strain SG-4G was purified and characterised. Further, this hydrolase was found to be an esterase-type and was employed for enzymatic *in situ* bioremediation (Pandey et al., 2010).

The 2-aminobenzimidazole is metabolised by a hydroxylase to 2-hydroxybenzimidazole. The furanyl ring of this intermediate is cleaved by a flavin-dependent monooxygenase to form benzene-1,2-diamine, which is further oxidised to catechol by benzoate-1,2-dioxygenase and mineralised. Carbendazim is a highly nitrated compound and serves as a sole nitrogen source (Singh et al., 2016; Long et al., 2020; Figure 3). In *Rhodococcus* sp. CX-1, the 2-hydroxybenzimidazole formed can alternatively undergo dihydroxylation of the aromatic ring followed by ring-cleavage by the action of an extradiol dioxygenase. These pathways have been proposed on the basis of whole-cell biotransformation, genome annotation and transcriptome analysis in strain CX-1 (Long et al., 2020; Figure 3).

#### Propoxur

Propoxur [(2-Propan-2-yloxyphenyl) *N*-methylcarbamate; Baygon] is an insecticide, acaricide and molluscicide. It is highly soluble in organic solvents and moderately soluble in water (Khalaf et al., 1993). Propoxur degradation pathway has been recently elucidated in the consortia SP1 consisting of *Pseudaminobacter* sp. SP1a and *Nocardioides* sp. SP1b, based on spent media analysis and whole-cell biotransformation studies (Kim et al., 2017). The strain SP1a harbours carbamate hydrolase, which catalyses the hydrolysis of propoxur to 2-isopropoxyphenol, methylamine and CO_2_. The methylamine generated is utilised as a C and N source by strain SP1a. Whereas the 2-isopropoxyphenol was proposed to be utilised through the formation of catechol by the strain SP1b (Kim et al., 2017; Figure 3). Earlier, propoxur hydrolase activity has been reported in the cell-free extract of a propoxur degrading *Pseudomonas* (Kamanavalli and Ninnekar, 2000).

#### Fenobucarb

Fenobucarb [(2-Butan-2-ylphenyl) *N*-methylcarbamate; BPMC] is widely used in rice fields and has been found to contaminate surface as well as ground water. Fenobucarb degradation pathway is partially elucidated, with only the carbamate linkage hydrolysis products, 2-*sec*-butylphenol and methylcarbamic acid, being reported. 2-*sec*-Butylphenol is further completely metabolised (Kim et al., 2014; Ufarté et al., 2017). A novel carboxylesterase, CE_Ubrb, recently identified by metagenomic analysis, was shown to carry out the hydrolysis of fenobucarb (Ufarté et al., 2017). Although not in fenobucarb degrading bacteria, 2-*sec*-butylphenol degradation pathway has been elucidated from other isolates like *Pseudomonas* sp. strain MS-1 where 2-*sec*-butylphenol is hydroxylated to form 3-*sec*-butylcatechol, which undergoes *meta*-cleavage and hydrolysis to yield aliphatic intermediates 2-hydroxypent 2,4-dienoic acid and 2-methylbutyric acid, thus acting as a carbon source (Toyama et al., 2010; Figure 3).

#### Phenmedipham

Phenmedipham, [3-(Methoxycarbonyl amino)phenyl *N*-(3-methylphenyl) carbamate; PMP] is a broad-spectrum herbicide that acts by disrupting the photosynthesis electron transport. Being hydrophobic, it gets adsorbed onto soil particles, leading to its accumulation. Besides plants, it also affects a broad range of non-target organisms like birds, fish and molluscs (Kamrin, 1997). The phenmedipham degradation pathway was recently deciphered in *Ochrobactum anthropi* NC-1 (Pujar et al., 2019). The pathway is initiated by hydrolysis catalysed by phenmedipham hydrolase into methyl *N*-hydroxyphenyl carbamate, *m-*toluidine and CO_2_. The former product undergoes a dealkylation reaction to yield 4-aminophenol, which is not further metabolised. The second product, *m-*toluidine undergoes oxidative deamination to form 4-methylcatechol. Ring-cleaving 4-methylcatechol 1,2-dioxygenase converts it into 2-hydroxy-5-methyl-6-oxohexa-2,4-dienoate which enters into central carbon pathway. Compared to glucose grown cells, phenmedipham grown strain NC-1 showed high level of activity of phenmedipham hydrolase (PheH), 4-methylcatechol 1,2- dioxygenase and *m-*toluidine dioxygenase enzymes in the cell-free extract (Pujar et al., 2019; Figure 3). Apart from strain NC-1, the phenmedipham hydrolase, Pcd from *Arthrobacter oxydans* P52 has been purified and characterised. This hydrolase was found to be an esterase type of enzyme and showed narrow substrate specificity with respect to phenmedipham (Pohlenz et al., 1992).

#### Methomyl

**FIGURE 4:**
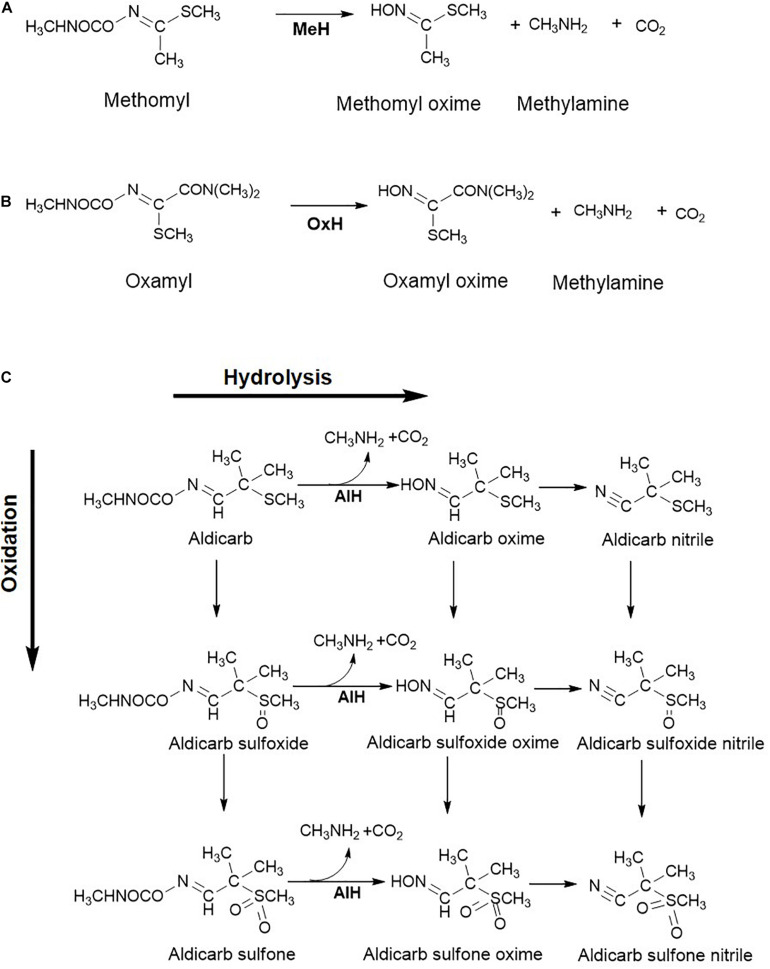
Metabolic steps involved in the oxime-based carbamate pesticide degradation: **(A)** Methomyl; **(B)** Oxamyl; and **(C)** Aldicarb. Various enzymes reported for biotransformation are: Methomyl hydrolase, MeH; Oxamyl hydrolase, OxH; Aldicarb hydrolase, AlH. Metabolism of aldicarb involves the hydrolytic route which results in the formation of oximes and upon further dehydration to nitriles, the oxidative route results in the formation of sulfones *via* sulfoxides. Further, metabolic routes for these pesticides are not elucidated in detail.

Methomyl [Methyl *N*-(methylcarbamoyloxy) ethanimi dothioate] is a carbamate family oxime pesticide which is water soluble and found to be widely distributed in the ecosystem (Lin et al., 2020). The methomyl degradation pathway has been elucidated for the consortia consisting of *Aminobacter* sp. MDW-2 and *Afipia* sp. MDW-3. Strain MDW-2 hydrolyses methomyl to methomyl oxime, methylamine and CO_2_ (Zhang C. et al., 2017). From strain MDW-2, the methomyl hydrolase, AmeH, an amidase type of enzyme has been purified and catalyses hydrolysis of methomyl to an unstable intermediate and methylamine. The former undergoes spontaneous conversion to methomyl oxime (Jiang et al., 2020b). Methylamine is utilised as a carbon and nitrogen source by MDW-2. The methomyl oxime generated is then utilised by strain MDW-3, although no intermediates were detected (Figure 4A).

#### Oxamyl

Oxamyl [Methyl 2-(dimethylamino)-*N*-(methylcarbamoyloxy)-2-oxoethanimido thioate], a carbamate nematicide, is highly susceptible to alkaline hydrolysis. Rousidou et al. reported the degradation pathway of oxamyl in four *Pseudomonas* strains where oxamyl was hydrolysed to oxamyl oxime and methylcarbamic acid (Rousidou et al., 2016). The latter, being unstable, forms CO_2_ and methylamine spontaneously. The gene *cehA*, encoding the oxamyl hydrolase, was detected in all four strains and its transcription was found to be 120–140 times higher in the oxamyl-amended cultures as compared to succinate-grown cells. Oxamyl oxime was reported to accumulate in the spent medium. The generated oxime is less toxic than the parent compound, therefore making hydrolysis of oxamyl an essential step in detoxification of this hazardous pesticide (Rousidou et al., 2016). A similar oxamyl degradation route has been proposed for *Micrococcus luteus* OX (Mohamed, 2017; Figure 4B).

#### Aldicarb

Aldicarb [(*E*)-(2-Methyl-2-methyl sulfanylpropylidene) amino *N*-methylcarbamate] is an insecticide and nematicide that is moderately soluble in water and is highly toxic. Bacterial transformation of aldicarb is known to proceed *via* the oxidative and hydrolytic route and bacteria utilise this carbamate as a sole carbon and nitrogen source (Figure 4C; Kazumi and Capone, 1995; Fareed et al., 2017). The former route involves oxidation of sulfur to yield aldicarb sulfoxide and further, aldicarb sulfone. These oxidation products are toxic. On the other hand, the hydrolytic pathway involves the hydrolysis of the carbamate linkage in aldicarb and the oxidation products, to form *N*-methylcarbamic acid and the respective oximes. These oximes further undergo dehydration to form the respective nitriles (Kazumi and Capone, 1995). Aldicarb hydrolase activity has been detected in the cell-free extract of *Enterobacter cloacae* strain TA7 (Fareed et al., 2017). The products of the hydrolytic pathway are less toxic than the parent compound (Figure 4C).

### Carbamate Pesticides as the Nitrogen Source

**FIGURE 5:**
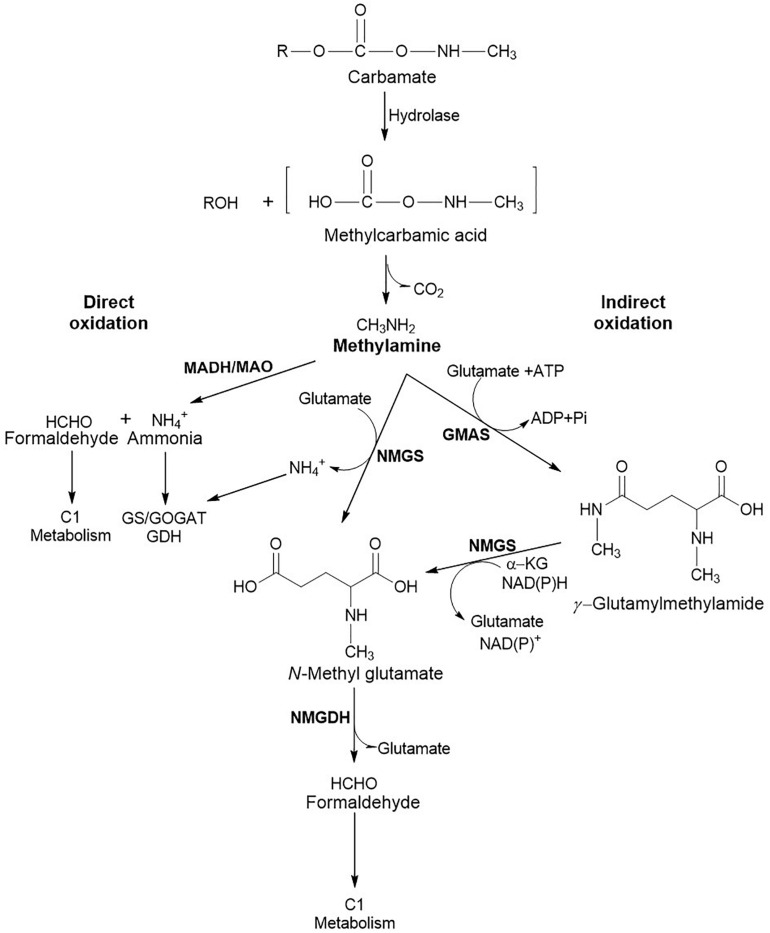
Metabolic routes involved in methylamine utilisation. The direct oxidation route involves MADH, Methylamine dehydrogenase in Gram negative and MAO, Methylamine oxidase in Gram positive bacteria. Indirect oxidation involves enzymes like NMGS, *N*-Methylglutamate synthase; GMAS, Glutamylmethylamide synthetase; and NMGDH, *N*-Methylglutamate dehydrogenase. Ammonia generated is utilised through the Glutamine synthetase/Glutamine oxoglutarate aminotransferase (GS/GOGAT) or Glutamate dehydrogenase (GDH) route, whereas formaldehyde is utilised through C1 metabolic route.

In addition to serving as the carbon source, carbamate pesticides are also utilised as the sole source of nitrogen. The enzymatic hydrolysis of the carbamate ester bond results in the formation of methylcarbamic acid which spontaneously breaks down into methylamine and CO_2_ (Rousidou et al., 2016). Methylamine can also be formed directly upon hydrolysis by an amidase as observed in the case of methomyl degradation (Jiang et al., 2020b). The ability to utilise methylamine as a carbon and nitrogen source is observed in methylotrophs and non-methylotrophs (Bicknell and Owens, 1980; Anthony, 1982). Bacterial utilisation of methylamine involves either direct or indirect oxidation routes (Figure 5). Direct oxidation of methylamine to formaldehyde and ammonia is carried out by a methylamine dehydrogenase (MADH) encoded by the conserved *mau* gene cluster in Gram negative bacteria and methylamine oxidase (MAO) in Gram positive bacteria (Chistoserdova, 2011). Whereas, indirect oxidation route involves amino acid glutamate to generate *N*-methylglutamate (NMG) either directly or through the intermediate *γ*-glutamylmethylamide (GMA). NMG is generated by the reaction of glutamate and methylamine, catalysed by NMG synthase (NMGS) and ammonia is released as a by-product. NMG formed is oxidised to formaldehyde and glutamate by NMG dehydrogenase (NMGDH). GMA synthetase (GMAS) catalyses the synthesis of GMA using methylamine and glutamate as substrates in presence of ATP. The GMA generated is then acted upon by NMGS to form NMG in the presence of α-ketoglutarate and NAD(P)H (Figure 5; Kamini et al., 2018a).

**FIGURE 6:**
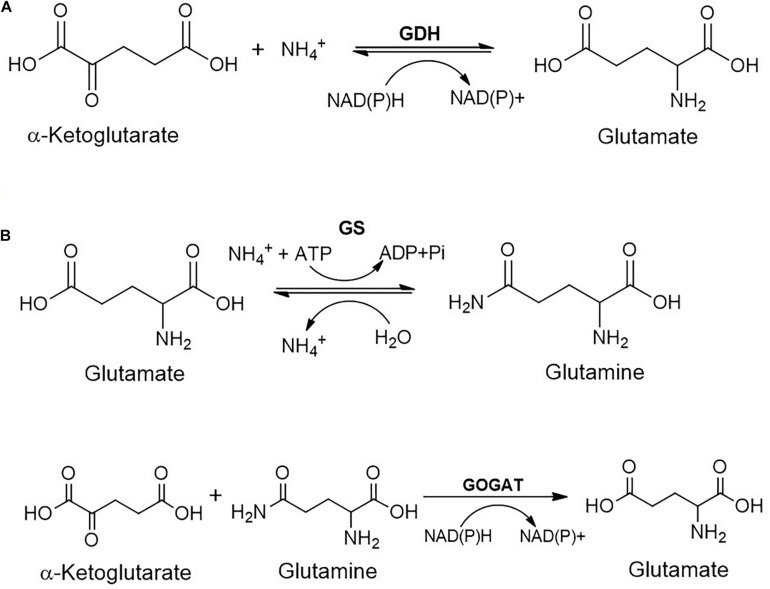
Metabolic fate of ammonia generated during the carbamate metabolism: **(A)** Glutamate dehydrogenase (GDH); or **(B)** Glutamine synthetase/Glutamine oxoglutarate aminotransferase (GS/GOGAT) route.

The ammonia generated can be assimilated either through glutamate dehydrogenase (GDH) or glutamine synthase-glutamate synthase (GS-GOGAT) route (Figure 6). The former route is low-affinity pathway and involves reaction of α-ketoglutarate with ammonia in the presence of NAD(P)H to form glutamate. GS-GOGAT route is high-affinity pathway and generates glutamine from glutamate and ammonia in the presence of ATP by GS. GOGAT yields two molecules of glutamate from glutamine and α-ketoglutarate in the presence of NAD(P)H (Figure 6; Hudson and Daniel, 1993; Ikeda et al., 1996; Reitzer, 2003).

**FIGURE 7:**
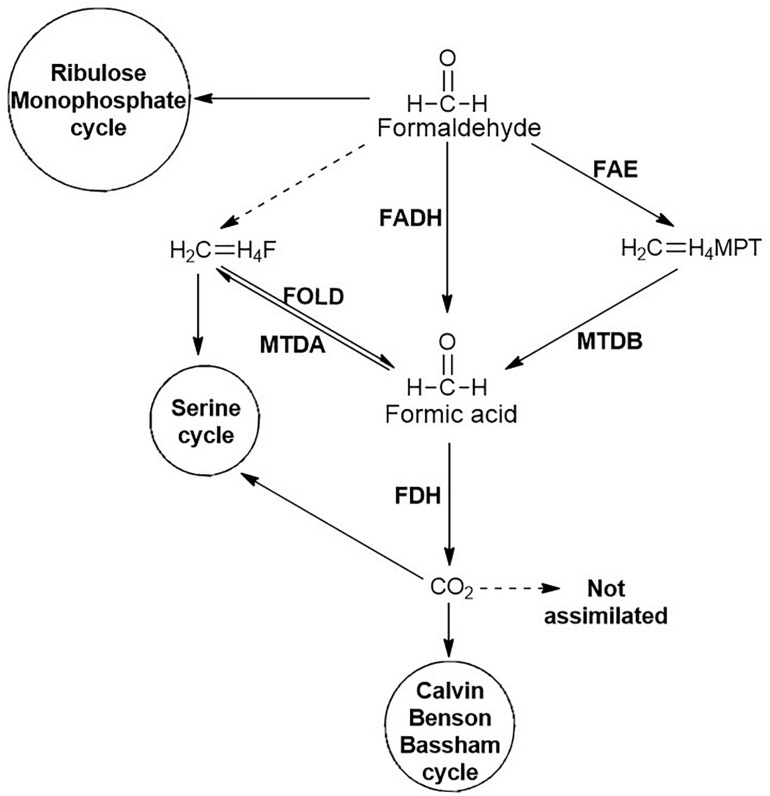
Fate of formaldehyde generated during carbamate degradation. Non-enzymatic reactions are represented by dashed lines whereas enzymatic reactions are represented by solid lines. Enzymes involved are: FADH, Formaldehyde dehydrogenase; FAE, Formaldehyde activating enzyme; FOLD, bifunctional Methylene-H4-Folate (CH_2_ = H_4_F) dehydrogenase-methylenetetrahydrofolate cyclohydrolase; MTDA, Methylenetetrahydrofolate (CH_2_ = H_4_F) dehydrogenase; MTDB, NAD(P)-dependent Methylene-tetrahydromethanopterin (CH_2_ = H_4_MPT) dehydrogenase; and FDH, Formate dehydrogenase.

Formaldehyde generated is either detoxified to CO_2_ or assimilated through ribulose monophosphate (RuMP) cycle (Figure 7). The detoxification is catalysed by formaldehyde dehydrogenase and formic acid dehydrogenase to yield CO_2._ Another route for the generation of CO_2_ involves the conjugation of formaldehyde to tetrahydromethanopterin, which undergoes oxidation to formic acid and further to CO_2_. The CO_2_ generated can be assimilated into the Calvin-Benson-Bassham (CBB) cycle. Alternatively, the formaldehyde can directly be conjugated with tetrahydrofolate and either enter into the serine cycle or yield formic acid (Figure 7; Chistoserdova, 2011).

## Fungal Metabolism of Carbamate Pesticides

**FIGURE 8:**
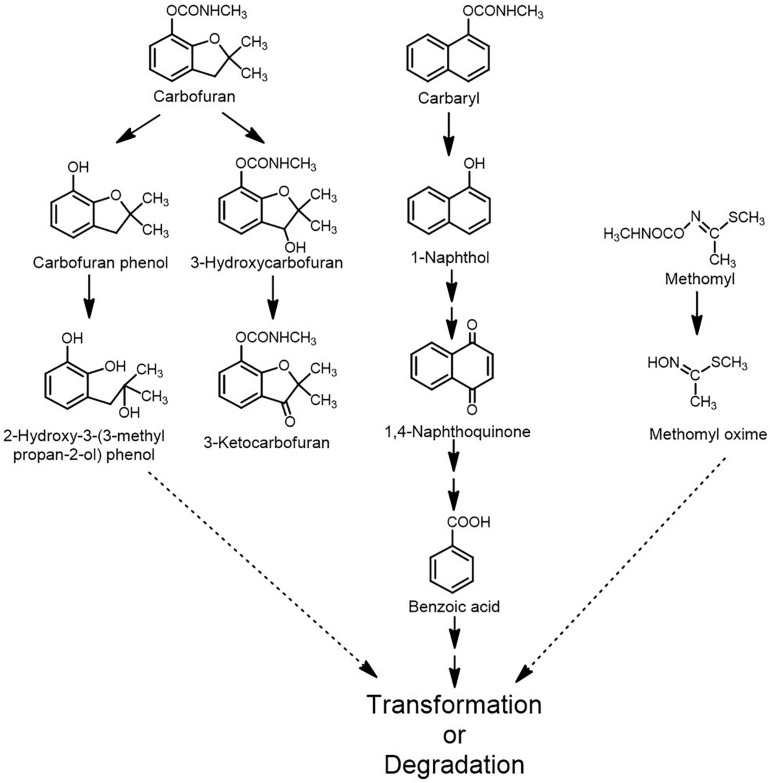
Metabolism/transformation of various carbamate pesticides by fungi.

The fungal metabolism of carbamates is an emerging field and various fungal strains belonging to the genera *Trichoderma*, *Pichia*, *Trametes*, *Aspergillus*, *Aschochyta*, *Xylaria*, *Acremonium*, *Gliocladium* and *Mucor* have been reported to either degrade or transform carbamate pesticides (Mustapha et al., 2019). In *Mucor ramannian*, carbofuran was hydrolysed to carbofuran phenol, which was further metabolised to 2-hydoxy-3-(3-methylpropan-2-ol)phenol or 7a- (hydroxymethyl)-2,2-dimethylhexahydro-6H-furo[2,3-b]pyran- 6-one and 3-hydroxycarbofuran-7-phenol (Figure 8; Seo et al., 2007). *Pichia anomala* HQ-C-01 has been shown to degrade carbofuran, Carbaryl and fenobucarb. The degradation intermediates identified for carbofuran were benzofuranol, 2-hydroxy-3-(3-methyl propan-2-ol) phenol and 3-hydroxycarbofuran (Yang et al., 2011). *Trametes versicolor* was reported to degrade carbofuran as well as the pyrethroid pesticides imiprothrin and cypermethrin. The only intermediate detected by spent media analysis for carbofuran degradation was 3-hydroxycarbofuran. In this strain, the non-specific monooxygenase cytochrome-P450 has been reported to play an important in carbofuran degradation (Mir-Tutusaus et al., 2014). Another strain of *T. versicolor* has also been reported to transform aldicarb, methomyl, methiocarb and carbofuran. The carbofuran was transformed into 3-hydroxycarbofuran and 3-ketocarbofuran by the action of cytochrome-P450 monoxygenase while no intermediates were reported for methomyl, aldicarb and methiocarb but laccase activity was proposed to be involved (Figure 8; Rodríguez-Rodríguez et al., 2017). *Aschochyta* sp. CBS 237.37 has been reported to degrade a mixture of the carbamates: carbofuran, Carbaryl, methiocarb, methomyl, oxamyl and propoxur. The spent media analyses showed carbofuran phenol and 3-hydroxycarbofuran as intermediates for carbofuran, methomyl oxime for methomyl and 1-naphthol for Carbaryl (Figure 8; Kaur and Balomajumder, 2019). *Acremonium* sp. has been shown to degrade Carbaryl and carbofuran and utilise them as a carbon and energy source. The carbofuran was hydrolysed to carbofuran-7-phenol and 3-(2-hydroxy-2-methylpropyl) benzene-1,2-diol. Whereas Carbaryl degradation proceeds through 1-naphthol and benzoic acid (Kaur and Balomajumder, 2020). Similarly, *Xylaria* sp. BNL1 has shown capability to degrade Carbaryl, which was hydrolysed to 1-naphthol and further degraded to 1,4-naphthoquinone and benzoic acid (Figure 8; Li et al., 2019). Three enzymes, Carbaryl esterase, laccase and cytochrome-P450 have been reported to participate in the degradation of Carbaryl from *Acremonium* and *Xylaria* sp. This pathway differs from the bacterial Carbaryl degradation pathway (Figure 3). CH from *Aspergillus niger* PY168, capable of hydrolysing Carbaryl, carbofuran, xylylcarb, metolcarb, propoxur, isoprocarb, fenobucarb, and aldicarb has been purified and characterised (Qing et al., 2003). Compared to bacterial metabolism, fungal metabolism is poorly understood, however, it indicates diverse routes of transformation/mineralisation.

## Genetics and Evolution of Carbamate Pesticide Degradation Pathways

The presence and persistence of toxic compounds like carbamate pesticides in the environment exerts a selection pressure on the microflora to evolve and adapt, while eliminating the sensitive microorganisms (Tien et al., 2013; Phale et al., 2019). Genetic variability is a major player in adaptation and survival of the population in response to exposure to toxic contaminants and is generated as a result of three processes: (i) small nucleotide changes, (ii) intragenomic shuffling, and (iii) horizontal gene transfer (HGT). The third mechanism allows organism to acquire new genetic material through mobile genetic elements (MGEs) like plasmids, transposons, genomic islands and integrative conjugative elements (Nojiri et al., 2004; Phale et al., 2019). Genes involved in the degradation of certain carbamate pesticides are found to be arranged as operons. These operons serve as an example of co-operative interaction between various genes that complement each other’s functions and are hence transferred together (Hall et al., 2020). Furthermore, the operonic arrangement aids in transcriptional regulation of degradative genes under the same promoter, thus fine-tuning their expression (Phale et al., 2019).

Apart from HGT, adaptations at molecular and cellular level play an important role in the evolution and optimisation of degradation traits. At the molecular level, enzyme promiscuity is a major factor that paves way for the evolution of enzymes that carry out novel reactions (Glasner et al., 2020). Certain enzymes might show broad substrate specificity and act on novel compounds to convert them into less toxic intermediates. Such non-specific activity mitigates the toxicity of the compound, thus enhancing the fitness and survivability of the organism in a contaminated niche. Due to the advantage they confer to the host strain, these enzymes undergo positive selection and over a period become efficient and specific in their action (Glasner et al., 2020). Among other strategies, compartmentalisation of enzymes prevents the interaction of toxic metabolic intermediates with cellular components, thus enhancing the efficiency of degradation (Kamini et al., 2018b).

### Genetics of Carbamate Degradation

#### Genetics of Carbon Metabolism

The genes encoding carbamate degradation enzymes are reported to be located either on the chromosome, plasmid or both. For example, the carbendazim degradation genes in *Rhodococcus* sp. CX-1 are localised on the plasmid as well as on the chromosome. The plasmid II harboured a gene cluster encoding carbendazim hydrolase (*mheI*), 2-hydroxybenzimidazole hydroxylase (*hdx*) as well as 2,6,7-trihydroxybenzimidazole dioxygenase (*edoB1B2C*; Table 2). The chromosome harboured a 2-aminobenzimidazole hydroxylase (*hdx*), 2,6,7-trihydroxybenzimidazole dioxygenase (*edoA*), 2-benzimidazolone monooxygenase (*mno*), benzoate-1,2-dioxygenase (*benA*) and the catechol degradation *catABC* gene cluster (Long et al., 2020). Another example is the Carbaryl degrading *Arthrobacter* sp. RC100, where the plasmid pRC1 encodes CH, while pRC2 encodes the enzymes for conversion of 1-naphthol to gentisic acid. The remaining enzymes for utilisation of gentisic acid are encoded by the chromosome (Hayatsu et al., 1999; Table 2).

**TABLE 2:** Plasmids involved in the degradation of carbamate pesticides.

**Organism**	**Plasmid(s), size (kb)**	**Associated metabolic properties**	**References**
*Sphingomonas* sp. CF06	pCF01, pCF02, pCF03, pCF04, pCF05 (5.5)	Enzymes involved in carbofuran mineralisation are encoded collectively by five plasmids. The role of individual plasmid is not clear.	Feng et al., 1997
*Achromobacter* sp. WM111	pPDL11 (100)	Encodes the carbofuran hydrolase, Mcd	Tomasek and Karns, 1989
*Sphingomonas* sp. TA	pCTOO1 (100)	Encodes the enzymes involved in carbofuran mineralisation	Ogram et al., 2000
*Sphingomonas* sp. CD	pCD2 (100)	Encodes the enzymes involved in carbofuran mineralisation	
*Rhizobium* sp. strain AC100	pAC200 (25)	Encodes the Carbaryl hydrolase, CehA	Hashimoto et al., 2002
*Arthrobacter* sp. RC100	pRC1 (110)	Encodes the Carbaryl hydrolase, CahA	Hayatsu et al., 1999
	pRC2 (120)	Encodes the enzymes for conversion of 1-naphthol to gentisic acid	
*Klebsiella oxytoca*	Two plasmids (100, 5.46)	Encodes the carbendazim hydrolase, MheI and other enzymes involved in carbendazim degradation	Alvarado-Gutiérrez et al., 2020
Strain NJ-D1	≥ 1 plasmid	Encodes the Carbamate hydrolase, CehA and other enzymes involved in fenobucarb degradation	Kim et al., 2014
*Stenotrophomonas maltophilia* M1	pMb (5)	Encodes the enzymes involved in methomyl degradation	Mohamed, 2009
Strain ER2	pER2a (120)	Encodes the carbamate hydrolase, Mcd for the degradation of carbofuran, Carbaryl and propoxur	Topp et al., 1993
*Arthrobacter oxydans* P52	pHP52 (41)	Encodes the phenmedipham hydrolase Pcd	Pohlenz et al., 1992
*Rhodococcus* sp. CX-1	Plasmid 2 (288)	Encodes the carbendazim amidohydrolase MheI and the extradiol dioxygenases involved in carbendazim mineralisation	Long et al., 2020
*Pseudomonas putida* XWY-1	pXWY (395)	Encodes the enzymes involved in Carbaryl mineralisation *via* gentisate route	Zhu et al., 2019

Carbamate degradation genes are often found to be present on plasmids along with insertion elements, therefore increasing the frequency of transfer of the degradation property through HGT. The carbofuran degrader, *Sphingomonas* sp. CF06 harbours five plasmids out of which three have a number of active IS elements. The plasmids pCF01 and pCF02 harboured IS1488, IS1487 and IS1412, whereas pCF03 has only IS1488. Moreover, four of them, pCF01, pCF02, pCF03 and pCF04, share a high sequence identity, indicating gene duplication (Table 2; Feng et al., 1997). The presence of active IS elements, along with gene duplication which indicates the lack of a positive regulatory system, suggests that this strain is still in an intermediate stage of evolution. Similar plasmid systems harbouring the insertion element IS1412 have been reported in the carbofuran degraders *Sphingomonas* sp. CD and *Sphingomonas* sp. TA (Ogram et al., 2000). Furthermore, degradation genes for other carbamate pesticides like carbendazim, carbofuran, Carbaryl, phenmedipham, fenobucarb, propoxur and methomyl, amongst others, have been found to be localised on the plasmid DNA (Table 2).

**FIGURE 9:**
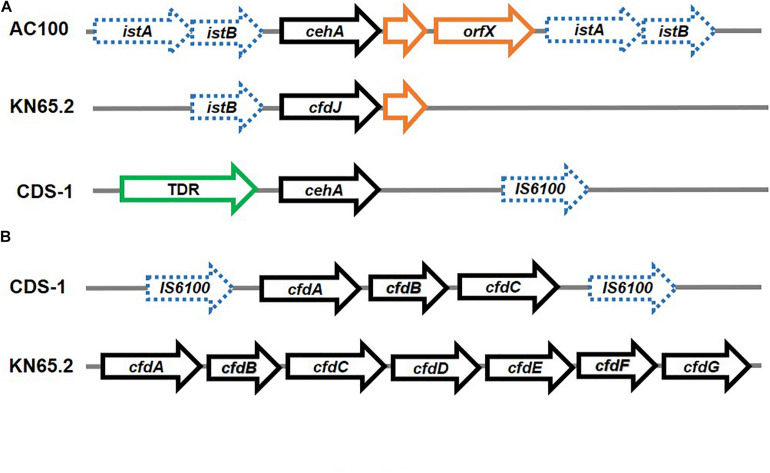
Organisation of genes involved in Carbaryl and carbofuran degradation: **(A)** Carbaryl hydrolase from *Rhizobium* sp. AC100 and carbofuran hydrolase genes from *Novosphingobium* sp. KN65.2 and *Sphingomonas* sp. CDS-1 – the flanking insertion elements have been depicted as dashed blue arrows whereas the pathway genes are depicted as black solid arrows, whereas ORFs have been depicted in orange and other associated structural genes (TonB dependent receptor; TDR) have been depicted in green and **(B)** operonic arrangement of genes (depicted as black solid arrows) for carbofuran degradation in *Novosphingobium* sp. KN65.2 and *Sphingomonas* sp. CDS-1. The insertion elements have been depicted as dashed blue arrows.

In few strains, all carbamate degradation genes are found to be localised on the chromosome. For example, the carbofuran-phenol degradation genes in *Novosphingobium* sp. strain KN65.2 and *Sphingomonas* sp. strain CDS-1 are arranged as *cfd* operons, while the gene encoding hydrolysis of carbofuran to carbofuran phenol (*cehA*) is present separately (Yan et al., 2018; Figure 9). The initial hydrolysis of carbofuran to carbofuran phenol in both the strains is carried out by homologues of the carbamate hydrolase CehA of *Rhizobium* sp. AC100. In strain AC100, *cehA* is flanked on both sides by insertion element-like sequences *istA-istB*, as well as an *orf* downstream, forming the transposon *Tnceh*. An identical gene arrangement is observed for the *cehA* encoding CH in *Rhodococcus* sp. X9 (Zhou et al., 2020). In strain KN65.2, *cehA* (also known as *cfdJ*) is flanked by a truncated *istB* upstream and a short *orf* downstream. Whereas in strain CDS-1, a Ton-B dependent receptor is present upstream of the *cehA* gene and IS6100 is present downstream. Strain KN65.2 also harbours the operon *cfdABCDEFGH*, encoding the complete carbofuran mineralisation pathway as well as a putative regulator. However, these genes are not part of an MGE. Whereas, the genes for carbofuran phenol transformation in strain CDS-1 are arranged as *cfdABC* operon, which encodes proteins for hydroxylation of carbofuran-phenol and regulation. This operon is bordered by IS6100 elements on both sides, indicating an MGE (Yan et al., 2018; Figure 9).

**FIGURE 10:**
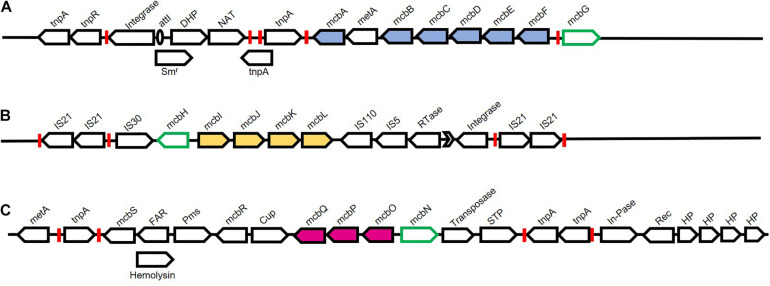
Chromosomal organisation of genes associated with Carbaryl degradation from *Pseudomonas* sp. strain C5pp. Pathway genes are depicted as filled arrows while black open arrows depict mobile genetic elements and associated genes. Green open arrows depict putative regulators. Various inverted repeats are depicted as red boxes. **(A)** Upper pathway genes encode enzymes involved in the conversion of Carbaryl to salicylate. Genes and protein encoded are Transposase (*tnpA* and *tnpR*); Integrase; *attI* site (unfilled oval); Streptomycin resistance (Sm^*r*^); Dihydropterate synthetase (DHP); *N*-Acetyltransferase (NAT); *tnpA*, Transposase; *mcbA*, Carbaryl hydrolase; *metA*, conserved protein; *mcbB*, 1,2-Dihydroxynaphthalene dioxygenase; *mcbC*, 1-Naphthol 2-hydoxylase; *mcbD*, 2-Hydroxychromene 2-carboxylate isomerase; *mcbE*, *Trans*-*o*-hydroxybenzylidene pyruvate hydratase-aldolase; *mcbF*, Salicylaldehyde dehydrogenase; *mcbG*, LysR family regulator. **(B)** Middle pathway genes encode enzymes involved in the conversion of salicylate to gentisate. Genes and protein encoded are: IS21 insertion elements flanked by inverted repeats; IS30 insertion element; *mcbH*, transcriptional regulator NahR; *mcbI*, Ferredoxin reductase; *mcbJ*, Salicylate 5-hydroxylase large oxygenase component; *mcbK*, Salicylate 5-hydroxylase small oxygenase component; *mcbL*, Ferredoxin; IS110 and IS5 insertion elements; Rtase, Reverse transcriptase; group-II intron (Black arrow head); Integrase; IS21 insertion elements flanked by inverted repeats. **(C)** Lower pathway genes encode enzymes involved in the conversion of gentisate to central carbon pathway intermediates. Genes and protein encoded are : *metA*, conserved protein; *tnpA*, Transposase; *mcbS*, TetR family transcriptional regulator; FAR, Fusaric acid resistance; Hemolysin; Pms, Permease; *mcbR*, LysR family transcriptional regulator; Cup, Pirin and cupin2 superfamily related protein; *mcbQ*, Maleylpyruvate isomerase; *mcbP*, Fumaryl pyruvate hydrolase; *mcbO*, Gentisate dioxygenase; *mcbN*, LysR family transcription regulator; Transposase; STP, Serine/threonine phosphatase; *tnpA*, Transposase; In-Pase, Inositol phosphatase; Rec, Recombinase; HP, Hypothetical protein.

In *Pseudomonas* sp. C5pp, all Carbaryl metabolic genes were found to be localised on chromosome, as indicated by southern hybridisation and plasmid curing experiments (Singh et al., 2013). Genes were found to be organised into three distinct operons with respective transcriptional regulators spanning a 76.3kb region, referred to as the Supercontig-A. The operons encoded the following metabolic steps: conversion of Carbaryl to salicylic acid (upper operon), salicylic acid to gentisic acid (middle operon), and gentisic acid to central carbon metabolites (lower operon) (Trivedi et al., 2016; Figures 3, 10).

The Supercontig-A harboured 17 transposases, out of the total 42 present in the genome, making it a hotspot for genome alterations (Figure 10). Further, the upper and middle operons showed considerably lower GC content as compared to the overall genome, indicating a different ancestral origin. The three operons were present as three distinct genomic islands (Trivedi et al., 2016). The upstream region of the upper pathway genes harboured class-I integron features, exhibiting high identity with Tn6217. Other features associated with the upper pathway genes included transposase, 25bp left-end repeat, *att*I site, resistance to streptomycin and two additional genes, indicating a high probability of HGT. Similarly, the regulator-encoding *mcbG* was flanked by truncated transposases at both the left and right ends, exhibiting similarity to ISPa20 of IS3 family and ISPst7 of IS5 family, respectively (Trivedi et al., 2016; Figure 10). These partial transposase sequences might be a reminiscent of a functional integrative conjugative element, which has been lost due to decay linked recombination events. Members of the class-I transposon family have been frequently implicated in xenobiotic degradation. The catabolic transposon, TnC5ppsal, harbours the middle pathway genes and is an example of one such transposon. The genes *mcbIJKL* are flanked by IS21 family insertion elements, present in inverted orientation, which is probably responsible for the stability of the transposon. Further, this operon harbours transposases at the 3′ end that show a high degree of similarity to IS110 and IS5 family, reverse transcriptase, group-II intron D1-D4-2, and leucine-zipper class of integrase. The lower pathway genes, on the other hand are bordered by non-identical insertion elements showing sequence similarity with ISPa1635 and IS481 and are also hypothesised to be a part of class-I transposon (Trivedi et al., 2016; Figure 10).

Interestingly, the gene arrangement of the upper, middle and lower operons (encoding Carbaryl degradation) in *P. putida* XWY-1 was found to be similar to that of strain C5pp. Further, the role of the regulator McbG has been functionally characterised in strain XWY-1. The protein McbG was identified as a LysR-type regulator that activated the transcription of the *mcbBCDEF* cluster (encoding enzymes that convert 1-naphthol to salicylate) upon interaction with 1-naphthol, the hydrolysis product of Carbaryl (Ke et al., 2021).

#### Genetics of Nitrogen Metabolism

Even though the reports on detailed metabolic and genetic analysis of methylamine utilisation are scarce in carbamate degrading organisms, genes encoding the enzymes for both the direct and indirect routes (Figure 5) have been established in carbamate pesticide degrading organisms. For example, the carbofuran degrader, *Novosphingobium* sp. strain KN65.2 harbours the *mauBEDA* operon predicted to encode a putative amine dehydrogenase for oxidative deamination of primary amines. However, strain KN65.2 failed to grow on methylamine (Nguyen et al., 2014). The gene *mauA*, encoding methylamine dehydrogenase (MADH), has been detected in oxamyl degrading *Pseudomonas* strains which utilise methylamine as both carbon and nitrogen source. This gene showed high sequence similarity to the *mauA* ecoding methylamine dehydrogenase in *Methylobacterium extorquens* strain AM1 (Rousidou et al., 2016). This enzyme is reported to carry out direct oxidation of methylamine to ammonia and formaldehyde in Gram negative bacteria (Chistoserdova, 2011). However, in strain AM1 the *mau* gene cluster is flanked by IS elements and associated sequences, indicating the involvement of HGT in transfer of the methylamine degradation property (Vuilleumier et al., 2009).

The detailed genomic and metabolic analysis of methylamine utilisation pathway has been carried out in *Pseudomonas* sp. C5pp, a Carbaryl degrader, which utilises methylamine as a sole nitrogen source *via* the indirect route (Kamini et al., 2018a). The draft genome analysis of this strain revealed the presence of two operons involved in methylamine metabolism: the *gmas-mgsABC-amt* operon and the *purU-folD-mgdABCD* operon. The former encodes GMAS, NMGS and inner membrane methylamine transporter, respectively. Whereas the *purU* and *folD* genes encode enzymes catalysing the conversion of formaldehyde to formic acid *via* the formation of methylene-tetrahydrofolate, while the *mgdABCD* cluster encodes NMGDH (Kamini et al., 2018a). These two gene clusters are 37.7 kb apart. In comparison, the methylamine metabolism genes in *Methyloversatilis universalis* FAM5 were arranged as the cluster *mgdABCD-gmas-mgsABC* (Latypova et al., 2010). Similarly, in *Methylocella silvestris*, the cluster *gltB1B2B3-gmas-soxBDAG* encoded the enzymes NMGS (*gltB123*), GMAS (*gmas*) and NMGDH (*soxBDAG*; Chen Y. et al., 2010). In strain C5pp, genes for the direct oxidation pathway were absent, suggesting GMA/NMG pathway to be the primary route for methylamine utilisation. Enzyme activity and qPCR studies showed that the expression of these enzymes is upregulated in presence of methylamine and Carbaryl, thus confirming that the GMA/NMG is the primary route for methylamine metabolism. Furthermore, the ammonia utilisation pathway was examined in strain C5pp (Kamini et al., 2018a). In presence of methylamine or Carbaryl (nitrogen limiting conditions), ammonia assimilation takes place via the GS-GOGAT route whereas in presence of NH_4_Cl (nitrogen abundance), assimilation takes place through the glutamate dehydrogenase (GDH) route (Figure 6). Formaldehyde was oxidised to formic acid either directly by a dehydrogenase or through the formation of methylene tetrahydrofolate. The formic acid generated through the metabolism of methylamine is not utilised for biomass production and is instead, detoxified to CO_2_ (Figure 7; Kamini et al., 2018a).

### Enzyme Promiscuity as a Positive Selection Pressure

The CH, CehA is an esterase from *Rhizobium* sp. AC100. Besides Carbaryl, recently it has been shown to act on Carbofuran with low efficiency (Yan et al., 2018). Homologs of this hydrolase have been found to act on various other carbamate pesticides like propoxur, oxamyl and fenobucarb, amongst others (Kim et al., 2014, 2017; Rousidou et al., 2016). For example, the CaH of *Novosphingobium* sp. strain KN65.2 and *Sphingomonas* sp. strain CDS-1 are homologs of CehA, with a difference of one amino acid substitution (in strain CDS-1) and four amino acid substitution (in strain KN65.2). Although CehA from strain CDS-1 (L152) differs from strain AC100 (F152) by just one amino acid substitution, it shows a higher activity on Carbofuran, rendering this mutation crucial (Yan et al., 2018). Similarly, the CehA from strain KN65.2 (called CfdJ) differs by four amino acids, including the F152L substitution. The rest three substitutions, although not silent, did not have the same crucial impact as the F152L mutation. In comparison to CehA, CfdJ showed higher activity on aromatic carbamates like propoxur, fenobucarb and lower on aliphatic carbamates like oxamyl (Öztürk et al., 2016). Additionally, the presence of similar homologs of CehA have been identified in the oxamyl degrading *Pseudomonas* spp. strain OXA17, OXA18, OXA20 and OXA25. While CehA from strain OXA18 was identical to that from *Rhizobium* sp. AC100, the other three strains differed by one amino acid substitution. The CehA from OXA17, OXA20 and OXA25 showed a broad specificity for both oxime (oxamyl, methomyl and aldicarb) and aryl-methyl carbamates (Carbofuran and Carbaryl). The relaxed specificity could possibly be attributed to the single amino acid substitution. The CehA_OXA20_ was also divergent from CfdJ with phenylalanine (instead of leucine) being present at the crucial 152 position (Rousidou et al., 2016). Additionally, the presence of threonine (hydroxy amino acid) at positions 494 and 570 in CehA homologs has been reported to diminish the activity on monocyclic (isoprocarb and propoxur) and linear (oxamyl and aldicarb) carbamates (Jiang et al., 2021). Recently, CehA from *Rhizobium* sp. strain AC100 has been shown to catalyse the hydrolysis of physostigmine. The legume *Physostigma benenosum* is responsible for the production of this naturally occurring toxic carbamate. This plant forms a symbiotic relationship with *Rhizobium*, which harbours the physostigmine hydrolysing enzyme pCehA. Based on these analyses, it has been proposed that CehA probably evolved from the physostigmine-hydrolyzing pCehA due to the selection pressure imposed by the introduction and widespread use of Carbaryl. Overtime, the gene *cehA* was distributed into the environment due to HGT (as indicated by the association with MGEs), giving rise to specific CehA homologs (Jiang et al., 2021).

1NH from *Pseudomonas* sp. C5pp has been reported to show 47 % activity on 2,4-dichlorophenol as substrate and 55 % identity with 2,4-dichlorophenol 6-monoxygenase from *Paraburkholderia zhejiangensis* (Phale et al., 2019). It is interesting to note that although 1NH does not show activity on phenol, it can carry out the hydroxylation of 2,4-dichlorophenol (47 % efficiency). Furthermore, this enzyme accepts both 1-naphthol (K_m_ = 11.3 μM) and 2,4-dichlorophenol (K_m_ = 13.3 μM) with equal affinity (Trivedi et al., 2016). These observations suggest that 1NH probably evolved by acquiring the 2,4-dichlorophenol 6-monoxygenase gene through HGT followed by the mutations at the substrate binding pocket to catalyse the reaction with 1-naphthol as a substrate. The hydroxylase component CehC1 of the recently reported two-component 1NH, CehC1C2 from *Rhizobium* sp. X9 shows 58% similarity with the oxygenase component of two-component 4- nitrophenol 2-monooxygenase from *Rhodococcus* sp. PN1 and 64% activity on 4-nitrophenol as compared to 1-naphthol (Zhou et al., 2020).

### Periplasmic Localisation as a Strategy for Efficient Degradation

**FIGURE 11:**
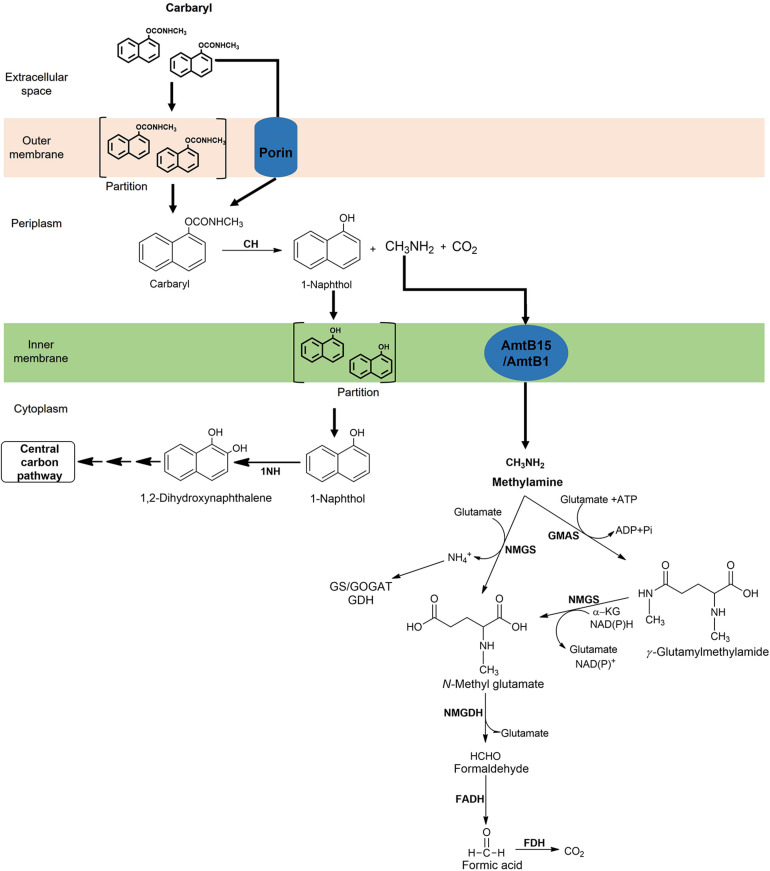
Compartmentalisation of Carbaryl degradation and methylamine metabolic pathway in *Pseudomonas* sp. C5pp.

During metabolism of certain xenobiotics, toxic/reactive intermediates are produced, which can cause damage to cytoplasmic components upon interaction. To escape toxicity, various bacteria have evolved to adopt a strategy wherein enzymes responsible for generation of such reactive intermediates are compartmentalised. For example, the hexachlorocyclohexane dehalogenases (LinA and LinB) from *Sphingomonas paucimobilis* UT26, quinohemoprotein alcohol dehydrogenase from the dichloropropanol-degrader *Pseudomonas putida* strain MC4 as well as various carbamate hydrolases (Arif et al., 2012; Phale et al., 2020). An N-terminal signal peptide sequence has been found to be present in CH from *Rhizobium* sp. strain AC100 and *Arthrobacter* sp. strain RC100 as well as in CaH of *Novosphingobium* sp. strain KN65.2 and *Achromobacter* sp. strain WM111 (Kamini et al., 2018b). It is proposed that these proteins are transported either through the “Sec” or “Tat” pathway, although the periplasmic localisation of these proteins has not been established experimentally (Öztürk et al., 2016). The CH from *Pseudomonas* sp. C5pp has been shown to be localised in the periplasm and has a N-terminal transmembrane domain region (Tmd; 73 amino acid long) followed by the signal peptide sequence (23 amino acids; Kamini et al., 2018b). Enzymes responsible for 1-naphthol and methylamine metabolism are found to be present in the cytoplasm. The action of the low affinity CH (K_m_ = 100 μM) generates 1-naphthol (which is a toxic as well as reactive phenolic compound) in the periplasm, which is transported across the inner membrane through partition and diffusion process into the cytoplasm, where it gets ring-hydroxylated by high affinity 1NH (K_m_ = 10 μM) to form 1,2-dihydroxynaphthalene, which is metabolised rapidly. Therefore, the cellular compartmentalisation in strain C5pp serves as an efficient adaptation strategy to prevent the interaction of 1-naphthol with the cytoplasmic macromolecules and machinery (Figure 11; Kamini et al., 2018b).

## Strategies for Optimising the Bioremediation of Carbamate Pesticides in the Environment

Physico-chemical methods like adsorption, photolysis, photocatalysis, advanced oxidation processes and cavitation are often employed for the *in situ* clean-up of carbamate pesticides (Table 3). Although the degradation process is quick, these techniques often generate toxic by-products, are inefficient and cost intensive (Parte et al., 2017). Microbial degradation often leads to complete mineralisation of these compounds, is cost-effective and eco-friendly. Degradation of xenobiotics, including carbamate pesticides, has been reported widely in various isolates, as described. However, use of these strains in bioremediation process poses limitations, for example, slow degradation rates, lack of complete degradation pathway in a single organism and carbon source utilisation hierarchy due to the presence of simple carbon sources, amongst others (Nielsen, 2001; Dvořák et al., 2017; Phale et al., 2020). Further, environmental factors like non-optimal pH and oxygen limitation also impact the survivability of these organisms. *In-situ* bioremediation procedures like bioaugmentation and/or bio-stimulation aid in enhancing degradation by optimising extrinsic factors and environmental conditions and/or by increasing the microbial load at the contaminated site (Mir et al., 2020). However, intrinsic cell-specific factors like incomplete metabolic pathways and carbon catabolite repression still limit the bacterium’s degradation ability. To overcome these limitations, two major approaches, consortia based-degradation and metabolic engineering can be employed either alone or in combination to enhance the degradation efficiency of these compounds (Bhatt et al., 2021a) Moreover, the past decade has witnessed the integration of metabolic engineering approaches with system biology, aiding in overcoming potential pitfalls by employing databases, bioinformatics and computational tools for prediction of thermodynamic feasibility and compound toxicity, vector design, flux balance analysis, gene and protein structure analysis, amongst others. “Omics” techniques have aided in obtaining a broader perspective on the genomic, metabolic, proteomic and transcriptomic aspects of cellular metabolism and degradation, thereby aiding in rational pathway design (Dvořák et al., 2017; Dangi et al., 2019; Jaiswal et al., 2019; Mishra et al., 2021a). Other approaches like adaptive laboratory evolution can further aid in fine-tuning these catabolic traits.

**TABLE 3:** Physico-chemical methods employed in the remediation of carbamates.

**Pesticide**	**Physicochemical method employed**	**Remarks**	**Intermediates detected/degradation route**	**References**
Carbofuran	Adsorption onto GAC F300 activated carbon	Maximum adsorption capacity of 96.15 mg of Carbofuran per g of adsorbent; equilibrium reached within 10–22 h	–	Salman and Hameed, 2010
	UV/H_2_O_2_ AOP	100% mineralisation in 3 h	2,2-dimethyl-2,3-dihydrobenzofuran-7-ol, 2-dimethyl-2,3-dihydrobenzofuran-3,7-diol, 7-hydroxy-2,2dimethyl benzofuran-3(2*H*)-one, pyrocatechol, formic acid, oxalic acid, acetic acid	Ibrahim and Şolpan, 2019
Oxamyl	Adsorption onto Silkworm faeces activated carbon (SFAC)	Removal of 99.48% oxamyl; equilibrium reached within 2 h	–	Mohammad and Ahmed, 2017
Carbaryl	Adsorption onto Surface molecularly imprinting polymer (SMIP)	Adsorption capacity of 41.14 mg/g; equilibrium reached within 20–40 min	–	So et al., 2018
	Photolysis (Solar)	10–60% degradation achieved depending upon the particle size and presence of organic matter	2-hydroxy-1,4-naphtoquinone and 1-naphthol	Siampiringue et al., 2015
	UV/O_3_ AOP	Complete degradation of 40 mg/L Carbaryl at 75 s ozonation time	4-(1-hydroxy-2-(methylamino) propyl) phenol, naphthalen-2-ol, 2-(hydroxymethyl) benzoic acid, 2-(2- ethoxy ethoxy) acetic acid, 4-(1-hydroxy-2-(methylamino)-propyl) phenol, (Z)-2-(methoxyimino) heptanoic acid, (Z)-2-(methoxyimino)-3-methylbutanoic acid, 2-formamidoacetic acid, 2-amino-3-hydroxybutanoic acid, citric acid, malonic acid, acetic acid, formic acid	Ibrahim and Şolpan, 2020
	Electro-Fenton process	90% total organic carbon removal within 2 h	1-Naphthol, Naphthohydroquinone, 1,4-Naphthoquinone, 1,4-Naphthalene dione-2-hydroxy, 1,4-Naphthalene dione-5-hydroxy, Phtalic anhydride, Phthalic acid-*O*-yl *N*-methylcarbamate, Phthalic acid, Phtalaldehydic acid, oxalic acid, acetic acid, formic acid, succinic acid	Çelebi et al., 2015
Methomyl	UV/TiO_2_ AOP	100% removal within 45 min	Methomyl oxime, acetonitrile, glycolic acid, oxalic acid, formic acid	Tamimi et al., 2006
	Ultrasound cavitation coupled with fenton and/or photo-fenton process	28.7% removal with only ultrasonic cavitation in 72 min; 100% removal when coupled with fenton or photo-fenton in 18 and 9 min, respectively	–	Raut-Jadhav et al., 2016
Carbendazim	Photo-Fenton process	96% removal of carbendazim within 15 min	2-aminobenzimidazole, benzimidazole isocyanate and monocarbomethoxyguanidine	da Costa et al., 2019
Pyraclostrobin	Hydrolysis and photolysis (under sunlight and UV light) in aqueous solution and paddy water	Half-life of 1.32–2.96 h in UV light; 3.69–11.2 h in sunlight depending upon the pH	Hydrolysis by loss of *N*-methoxy group and *N-*methyl formate, followed by hydroxylation of pyrazol ring. Photolysis follows substitution of chlorine with hydroxyl group, followed by either removal of the methoxy group or scission of 4-hydroxyphenyl and pyrazol bond.	Zeng et al., 2019

### Consortia for the Degradation of Carbamate Pesticides

Carbamate pesticides in soil and water can present as mixtures with varying concentrations. Therefore, a single bacterium is often limited in its capacity to degrade diverse range of these pesticides at excessive or varying concentrations. As compared to single strain, mixed bacterial cultures (consortium) depict better degradation and survivability due to sharing of metabolic burden/division of labour, resilience to environmental fluctuations, resistance to invasion by other species and prevention of “metabolic cliff” (Bhatt et al., 2021a). A large number of naturally occurring, carbamate degrading bacterial consortia have been reported from diverse environments like river biofilms (Chen et al., 2015), agriculture soil (Sharif and Mollick, 2013), pesticide disposal sites (Chapalamadugu and Chaudhry, 1991) and lake and salt marsh sediments (Kazumi and Capone, 1995). In some of these consortia, the metabolic and genetic aspects of degradation have been studied. Members exhibit metabolic synergism, that is, the metabolic intermediates produced by one isolate are mineralised by other members of the consortia. For example, the Carbaryl degrading consortium consisting of *Pseudomonas* spp. 50552 and 50581 exhibits metabolic co-operation. Strain 50581 hydrolysed Carbaryl to 1-naphthol, which was secreted into the medium and further utilised by the strain 50552 (Chapalamadugu and Chaudhry, 1991). In propoxur degrading consortium SP1, *Pseudaminobacter* sp. SP1a converts propoxur to 2-isopropoxyphenol, which is utilised by *Nocardioides* sp. SP1b as a carbon source (Kim et al., 2017). In a methomyl degrading consortium, *Aminobacter* sp. MDW-2 hydrolysed methomyl to methomyl oxime while *Afipia* sp. MDW-3 utilised methomyl oxime (Zhang C. et al., 2017). Similar synergistic interactions have been observed for a carbofuran degrading consortium consisting of *Arthrobacter* sp. and *Pseudomonas* sp. as well as another seven-member consortium (Singh et al., 1993; Mohapatra and Awasthi, 1997). Apart from naturally occurring consortia, “synthetic consortia” can be constructed by assembling specific bacterial isolates with complementary metabolic properties. For example, the consortium of *Escherichia coli* SD2 and *Pseudomonas putida* KT2440 pSB337 has been constructed for the degradation of organophosphate pesticide parathion. Strain SD2 hydrolysed parathion to *p*-nitrophenol, which was further mineralised by strain KT2440 pSB337 (Gilbert et al., 2003). Apart from metabolic co-operation, biosurfactant and siderophore production by isolates might contribute to the interactions arising between different members of the consortia for effective clean-up (D’Onofrio et al., 2010; Liang et al., 2018). Thus, the successful development of a synthetic consortia requires an understanding of microbial interactions, microbial communication (quorum sensing), dynamics of the community and behaviour of individual members (Bhatt et al., 2021a).

### Metabolic Engineering

Metabolic engineering involves directed construction of metabolic pathway and related traits using various genetic engineering tools (as well as by system biology approaches) (Bailey, 1991; Stephanopoulos and Vallino, 1991). This process has been employed for enhancing the degradation of major classes of pesticides like organophosphates (Zhang et al., 2016), organochlorines (Lan et al., 2014) as well as carbamates (Lan et al., 2006; Gong et al., 2016, 2018).

*P. putida* KT2440 has been engineered for the simultaneous degradation of chlorpyrifos and carbofuran by the co-expression of CaH (Mcd) from *Achromobacter* sp. WM111 and chlorpyrifos hydrolase (Mpd) from *Stenotrophomonas* sp. YC-1. Both genes were chromosomally integrated and expressed under the constitutive promoter J23119. The newly constructed strain, *P. putida* KTU-PGC hydrolysed chlorpyrifos to 3,5,6-trichloro-2-pyridinol and diethylthiophosphoric acid, and carbofuran to carbofuran phenol and methylamine (Gong et al., 2016). The metabolic versatility of *P. putida* KT2440 has further been broadened to simultaneously degrade organophosphates, pyrethroids and carbamates. The engineered strain, *P. putida* KTUe, harboured four degradation genes expressed under the J23119 promoter: methylparathion hydrolase (*mpd*) from *Stenotrophomonas* sp. strain YC-1, pyrethroid-hydrolyzing carboxylesterase (*pytH*) from *Sphingobium wenxiniae* strain JZ-1 and two carbamate degradation genes; CaH (*mcd*) from *Achromobacter* sp. strain WM111 and CH (*cehA*) from *Rhizobium* sp. strain AC100. Furthermore, genes for green fluorescent protein (GFP) and *Vitreoscilla* haemoglobin (VHB) were also introduced under the same expression system for real time monitoring and enhanced oxygen sequestration, respectively. The strain KTUe could simultaneously hydrolyse methylparathion, chlorpyrifos, fenpropathrin, cypermethrin, carbofuran, and Carbaryl, although the hydrolysis products were not utilised (Gong et al., 2018). The *cehA* gene, encoding CH in *Rhizobium* sp. strain AC100 has been functionally expressed and displayed on the surface of chlorpyrifos-degrader *Stenotrophomonas* sp. strain YC-1. A truncated version of the ice nucleation protein InaV from *Pseudomonas syringae* INA5 was used as an anchoring motif by fusing it with the CH (Yang et al., 2017). The degradation capacity of such genetically engineered isolates can further be exploited by using them as members of consortia.

The carboxylesterase B1 is a key enzyme that mediates resistance to organophosphates in mosquitoes and has the potential to act on carbamate pesticides. Lan et al. co-expressed organophosphate hydrolase gene (*opd*) from *Flavobacterium* sp. and carboxylesterase B1 gene (*b1*) from *Culex pipiens* into *Escherichia coli* strain BL21 (DE3) and checked the degradation of pirimicarb (a recalcitrant carbamate insecticide) along with parathion and deltamethrin by purified B1. Although B1 did not efficiently hydrolyse pirimicarb, it was proposed that active site optimisation is needed to enhance the degradability (Lan et al., 2006).

Plasmid or chromosome-based molecular biology tools can be employed for the expression of degradation genes. Replicating plasmids require a constant selection pressure, have highly variable copy number (Lin-Chao and Bremer, 1986; Paulsson and Ehrenberg, 2001) and impose a metabolic load due to expression of multiple copies of the gene (Mi et al., 2016). Therefore, chromosomal integration is a highly desirable approach for engineering metabolic pathways in the suitable host.

Furthermore, the presence of simpler carbon sources in the environment reduces the efficiency of removal of xenobiotics. To overcome this limitation, isolates like *Pseudomonas putida* CSV86 can be used as a metabolic engineering *chassis*. Strain CSV86 preferentially utilizes aromatic compounds over simple carbon sources like glucose and co-metabolizes aromatics with organic acids. This behaviour is unique and is not observed in other aromatic degrading Pseudomonads or bacteria (Basu et al., 2006). In addition, the aromatic degradation property is very stable in CSV86 as it is localised on the chromosome. Therefore, such isolate is an ideal host for the metabolic engineering (Phale et al., 2019, 2020). For example, strain CSV86 can be engineered to degrade Carbaryl preferentially over simple carbon source by expressing CH and 1NH from Carbaryl degrading strains.

Apart from introducing degradation genes, metabolic engineering can aid in enhancing the fitness of the microbial *chassis*. For example, deletion of 300 non-essential genes from the genome of *P. putida* KT2440 resulted in a strain with superior growth properties and improved physiological fitness. Moreover, the newly constructed strain EM383 had a higher NADPH/NADP^+^ ratio, resulting in better oxidative stress tolerance, which is a desirable property for aerobic biodegradation reactions (Martínez-García et al., 2014).

### Adaptive Laboratory Evolution

Nature is the largest and most efficient laboratory for evolving organisms. As a consequence of exposure to pesticides and disinfectants, bacterial populations have adapted and evolved the necessary metabolic pathways to overcome toxicity (Nojiri et al., 2004). In addition to the environment, this process of adaptation can also be experimented in the laboratory and is referred as adaptive laboratory evolution. This process involves fine-tuning degradation phenotype of a suitable microbe by exposing it to the target compound and observing the population over several generations (Dvořák et al., 2017). For example, the evolution of atrazine (a triazine herbicide) degrading *Pseudomonas* sp. ADP-1 was studied for 320 generations by subculturing on a liquid medium containing atrazine as the sole nitrogen source. Overtime, a new population that grew faster and degraded atrazine more rapidly, replaced the initial population. To explain this observation, it was hypothesised that the tandem duplication of *atzB* gene (encoding the second enzyme of the atrazine pathway) was responsible for the enhanced fitness (Devers et al., 2008). Similar gene duplication event was observed over 1000 generations in *Ralstonia* sp. strain TFD41 for 2,4-D (herbicide) degradation. The duplication of the *tfdA* gene (encoding a 2,4-D dioxygenase that catalyzes the first step of degradation) indicated that degradation capacity enhanced overtime by genotypic evolution. Furthermore, a 2.4 kb region in the chromosome was deleted, probably because the genes carried detrimental information or provided no selective advantage and contributed to the metabolic load (Nakatsu et al., 1998). In both cases, the duplications were reported to be mediated by insertion elements, indicating their role in gene duplication, thus allowing cells to utilise these toxic compounds more efficiently. Therefore, adaptive laboratory evolution can be applied to enhance and fine-tune the degradation efficiency of pesticides in natural or engineered isolates and holds great potential for application to the degradation of carbamate pesticides (Dvořák et al., 2017).

## Conclusion and Future Perspectives

Far excess use of carbamate pesticides has resulted in their dispersal across various compartments of the ecosystem. This has imposed a selection pressure on the microbiota to mitigate their toxicity by detoxification/transformation or mineralisation. Interestingly, the reported bacterial degradation pathways for various carbamates primarily involve hydrolases and oxido-reductases; and depict conserved metabolic themes such as the initial hydrolysis by either amidases or esterases, ring-hydroxylation of the hydrolysis products (for aryl carbamates), generation of corresponding dihydroxy compounds and subsequent ring-cleavage to aliphatic intermediates, which are funnelled into central carbon metabolism. Moreover, the generated methylamine is utilised as a sole carbon and nitrogen source. Enzymes involved in the degradation of various carbamates share a high degree of sequence similarity with each other, as observed in the case of CehA type hydrolases. Such sequence homology is also observed with enzymes involved in metabolism of structurally related compounds, as observed for 1-naphthol 2-hydroxylase and 2,4-dicholorophenol monooxygenase (55% identity at the amino acid level). This similarity points toward an evolutionary process manifesting itself by positive selection of promiscuous enzymes in a contaminated environment, working in tandem with horizontal gene transfer. To further validate this notion, the genes encoding carbamate degradation are often found to occur as operons and/or to be associated with mobile genetic elements like insertion elements, plasmids, genomic islands and transposases. Additionally, cellular compartmentalisation of the pathway enzymes (earlier reported for HCH dehalogenases and later for Carbaryl hydrolase) aids in enhancing the degradation efficiency of carbamates by preventing the interaction of toxic intermediates with cellular components. Although available, detailed analyses on the genetics, evolution as well as enzyme structure-function relationships of carbamate degradation are still limited (especially in comparison to other xenobiotics/pesticides) and pose a lacuna that needs to be fulfilled.

The *in-situ* bioremediation of carbamates poses a major challenge to the survivability and degradation capacity of the organism (even though laboratory scale degradation might be efficient). Metabolic engineering of the isolate can aid in overcoming these challenges by expression of the necessary degradative genes in a suitable *chassis* or by improving the physiological vigour *via* deletion of non-essential genes. Available reports of carbamate degradation pathway engineering have focussed on achieving the partial biotransformation of these compounds to their hydrolysis products instead of engineering of complete mineralisation pathways (Gong et al., 2016, 2018). Interestingly, degradation pathways of various aromatic compounds share similarity to the pathways reported for various aryl carbamates (for example, naphthalene and Carbaryl degradation pathways). Furthermore, the genetics and metabolism of aromatic compounds are well-studied in bacteria, making it convenient to identify potential metabolic nodes and genes for engineering aryl-carbamate mineralisation pathways. Apart from metabolic engineering, consortia-based remediation is an attractive alternative for enhancing *in situ* degradation and various naturally occurring consortia have been reported to efficiently degrade carbamates. Nevertheless, the degradation capacity of these consortia can further be enhanced by engineering the desirable bacterium. Construction of a “synthetic consortium” by assembling bacteria (which might be engineered) harbouring suitable pathways provides another desirable alternative. Such synthetic consortia have been constructed for clean-up of lignocellulosic biomass (Minty et al., 2013), cellulose (Tozakidis et al., 2016), organophosphates (Gilbert et al., 2003) as well as 2,4,6-tribromophenol (Li Z. et al., 2015), but not carbamates. To complement these strategies, systems biology and adaptive laboratory evolution serve to improvise and enhance the degradation potential of the bacterium. Therefore, metabolic engineering, in tandem with consortia-based degradation and adaptive laboratory evolution, holds great potential for overcoming the challenges associated with *in situ* bioremediation.

Although recent advancements in the field of carbamate degradation have aided in expanding our understanding of the underlying metabolic, evolutionary, biochemical and genetic mechanisms, various aspects still remain elusive:

(A) In order to gain a clear understanding of the structure-function relationship and the environmental behaviour of various degradation enzymes, it is essential to establish a putative evolutionary path. Although available (Trivedi et al., 2016; Zhou et al., 2020; Jiang et al., 2021), such analyses are very limited for carbamate degradation enzymes and pose a lacuna for future research.
(B) The detailed reports characterising the genetic, enzymatic, metabolic and biochemical aspects of carbamate degradation are limited. In most scenarios, only the transformation products are reported, instead of complete degradation pathways. Application of various “omics” approaches like genomics, metagenomics, transcriptomics, proteomics and metabolomics can aid in overcoming these limitations. Further, these techniques can provide valuable insights from “viable but non-culturable” bacteria (Mishra et al., 2021a).
(C) The current approaches for engineering carbamate degradation pathways have focussed on hydrolysis/detoxification aspects. Thus, the engineering of complete mineralisation pathways holds great potential. These approaches can also be combined with the engineering of degradation enzymes to enhance their efficiency. Efficiency of various esterases and hydrolases, involved in degradation of other xenobiotics, has been enhanced using mutagenesis and directed evolution approaches (Ma et al., 2013; Liu et al., 2019). Lastly, these approaches can aid in development of engineered “synthetic consortia.”
(D) The available literature on carbamate degradation focusses mostly on the utilisation of these pesticides as a carbon source, while detailed analyses on utilisation as nitrogen source are scarce. Thus, elucidation of the genetic, metabolic, transcriptomic and evolutionary aspects of nitrogen metabolism in carbamate degrading organisms holds great potential for future research.
(E) Available reports on carbamate degradation mostly focus on the bacterial metabolism of these pesticides, while the fungal metabolism of carbamates is poorly understood. Since remediation by fungal strains poses advantages like secretion of broad substrate-range extracellular enzymes, tolerance to higher concentrations of pollutants and enhanced bioavailability, understanding the genetic, metabolic and biochemical aspects of carbamate mycoremediation is cardinal.

## Author Contributions

All authors contributed equally toward conceptualisation, writing original draft, review and editing.

## Conflict of Interest

The authors declare that the research was conducted in the absence of any commercial or financial relationships that could be construed as a potential conflict of interest.

## Abbreviations

AChE: acetylcholinesterase
2,4-D: 2,4-dichlorophenoxyacetic acid
HPLC: high performance liquid chromatography
GC: gas chromatography
MS: mass spectrometry
ER: estrogen receptor
ROS: reactive oxygen species
CH: carbaryl hydrolase
CaH: carbofuran hydrolase
1NH: 1-naphthol 2-hydroxylase
S1H: salicylate-1-hydroxylase
S5H: salicylate-5-hydroxylase
HGT: horizontal gene transfer
MGE: mobile genetic element
GMA: *γ*-glutamylmethylamide
GMAS: *γ*-glutamylmethylamide synthetase
NMG: *N*-methyl glutamate
NMGS: *N*-methyl glutamate synthase
*NMGDH*: *N*-methyl glutamate dehydrogenase.
